# Supplementary material for: Single cell RNA sequencing of human liver reveals distinct intrahepatic macrophage populations
Source: Nat Commun. 2018 Oct 22;9:4383. doi: 10.1038/s41467-018-06318-7 (PMC6197289; doi:10.1038/s41467-018-06318-7)
Supplement: Supplementary file 1 — Supplementary Information [file 41467_2018_6318_MOESM1_ESM.pdf]

Single cell RNA sequencing of human liver reveals distinct intrahepatic macrophage populations

Sonya A. MacParland, Jeff C. Liu, Xue-Zhong Ma, Brendan T. Innes, Agata M. Bartczak, Blair K. Gage, Justin Manuel, Nicholas Khuu, Juan Echeverri, Ivan Linares, Rahul Gupta, Michael L. Cheng, Lewis Y. Liu, Damra Camat, Sai W. Chung, Rebecca K. Seliga, Zigong Shao, Elizabeth Lee, Shinichiro Ogawa, Mina Ogawa, Michael D. Wilson, Jason E. Fish, Markus Selzner , Anand Ghanekar , David Grant , Paul Greig, Gonzalo Sapisochin, Nazia Selzner, Neil Winegarden, Oyedele Adeyi, Gordon Keller, Gary D. Bader and Ian D. McGilvray

List of Supplementary Figures — Page

Supplementary Figure 1: Fractionation of the total liver homogenate samples into parenchymal and non-parenchymal cells leads to cell loss. 3
Supplementary Figure 2: Sample quality control. 5
Supplementary Figure 3: Heat map analysis showing the number of genes detected per cell. 6
Supplementary Figure 4: Cluster map analysis showing the 6 different mitochondrial transcript cut-offs. 7
Supplementary Figure 5: Additional cells which appear at various mitochondrial cut-offs. 8
Supplementary Figure 6: Doublet Filtering. 9
Supplementary Figure 7: Hematoxylin and eosin and Masson's trichrome staining of human liver parenchyma 10
Supplementary Figure 8: Similarity of human hepatocyte clusters to known mouse liver sinusoid layers. 12
Supplementary Figure 9: t-SNE plots showing the relative distribution of commonly expressed hepatocyte genes in the healthy liver. 14
Supplementary Figure 10: Pair-wise analysis of AFP+ and AFP- cells in all hepatocyte clusters (Clusters 1, 3, 5, 6, 14, 15). 15
Supplementary Figure 11: t-SNE plots showing the relative distribution of commonly expressed endothelial cell genes in the healthy liver. 17
Supplementary Figure 12: Pair-wise pathway analysis of liver endothelial cell populations 11 and 13 reveal few unique pathways activated. 18
Supplementary Figure 13: Pair-wise pathway analysis of liver endothelial cell populations 12 and 13 reveal unique pathways activated. 19
Supplementary Figure 14: t-SNE plots showing the relative distribution of commonly expressed macrophage genes in the healthy liver. 20
Supplementary Figure 15. Gating Strategy for Fig. 8d. 22
Supplementary Figure 16: Representative partitioning of lobules for immunohistochemistry (Figure 8 e-f). 23
Supplementary Figure 17: Distribution of macrophages by CD68 (general macrophage marker) vs. MARCO (non-inflammatory KCs). 25
Supplementary Figure 18: Cell cycle phase in each cluster. 26
Supplementary Figure 19: T and NK-like cell sub-analysis reveals additional distinct populations. 27
Supplementary Figure 20: Heat map analysis showing gene expression profiles of liver resident macrophages/Kupffer cells. 29
Supplementary Figure 21: Heat map analysis showing gene expression profiles of NK-like and B cells. 30

Supplementary Figure 22: Heat map analysis showing gene expression profiles of T cells......31
Supplementary Figure 23: Heat map analysis showing gene expression profiles of hepatic stellate cells.......32
Supplementary Figure 24: Heat map analysis showing gene expression profiles of endothelial cells and cholangiocytes.......33
Supplementary Figure 25: 10x Genomics Cell Ranger software summaries of unfiltered data from total liver homogenate 1......34
Supplementary Figure 26: 10x Genomics Cell Ranger software summaries of unfiltered data from total liver homogenate 2......35
Supplementary Figure 27: 10x Genomics Cell Ranger software summaries of unfiltered data from total liver homogenate 3......36
Supplementary Figure 28: 10x Genomics Cell Ranger software summaries of unfiltered data from total liver homogenate 4......37
Supplementary Figure 29: 10x Genomics Cell Ranger software summaries of unfiltered data from total liver homogenate 5......38

List of Supplementary Tables

Supplementary Table 1: Neurologically deceased liver donor details..........39

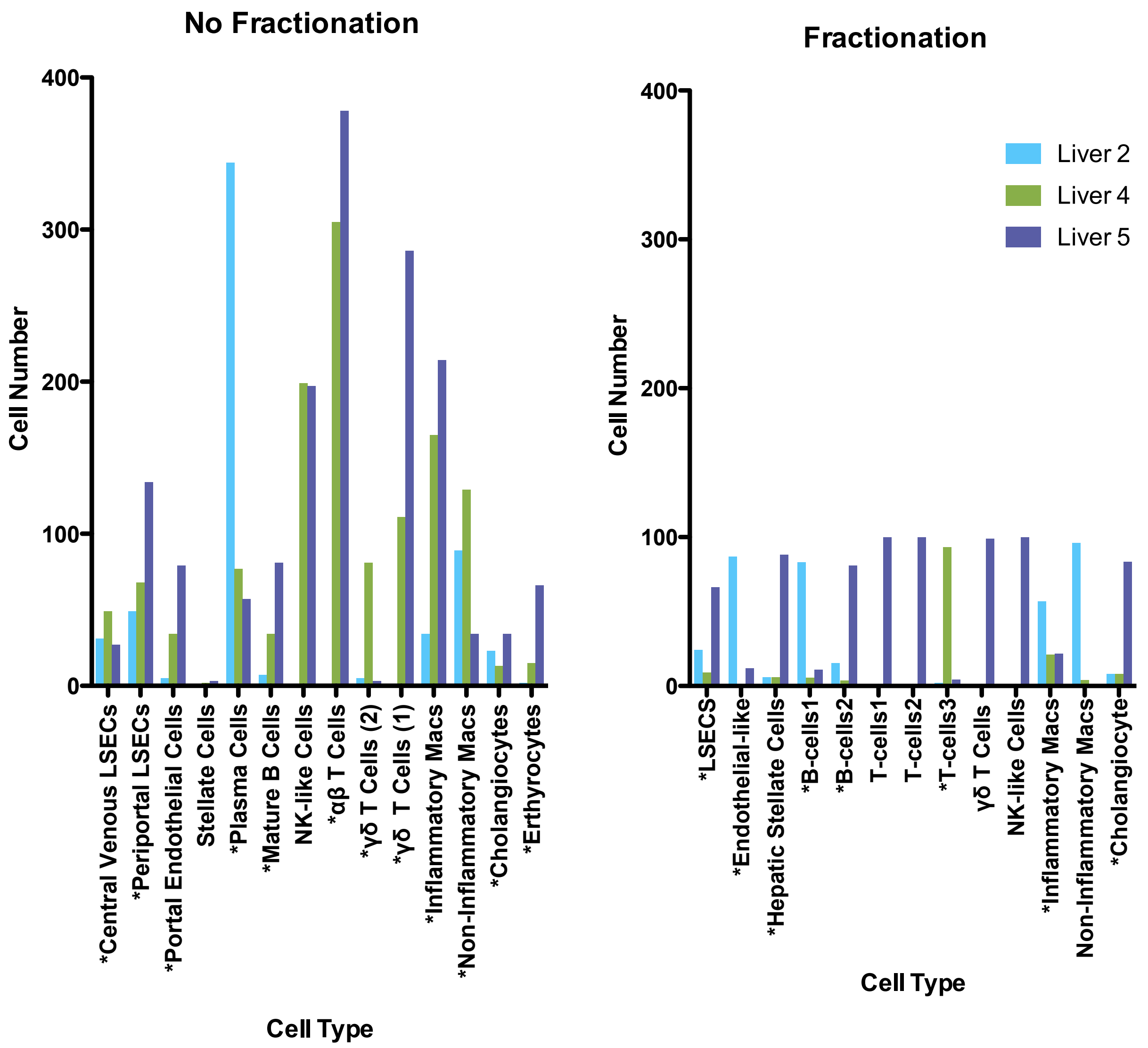

Supplementary Figure 1: Fractionation of the total liver homogenate samples into parenchymal and non-parenchymal cells leads to cell loss. Graphical summary of the proportion of cells that contributed to each cluster by liver sample. The left distribution is derived from the total liver homogenate. The right distribution is derived from non-parenchymal cells generated using a 50 x *g* centrifugation step and wash steps to remove hepatocytes. Asterisks denote clusters where cell from all three livers are represented. In both fractionated and unfractionated samples, 6000 cells were targeted for scRNA-seq analysis, however, the total number of viable cells analyzed after filtering out low quality cells was considerably less for fractionated cells, suggesting that these manipulations decreased overall cell viability.

A.

Patient	Viability of total liver homogenate by trypan blue exclusion	Cells in suspension targeted for sequencing (viable by trypan blue exclusion)	Filtered for Library Size > 1500	Filtered for Mitochondrial Proportion < 50%	Percent of cells in suspension passing QC filters
**P1TLH**	71%	6000	3672	1073	17.9%
**P2TLH**	49%	6000	1351	1331	22.2%
**P3TLH**	56%	6000	4345	3255	54.3%
**P4TLH**	90%	6000	2072	1532	25.6%
**P5TLH**	56%	6000	5653	1764	29.4%

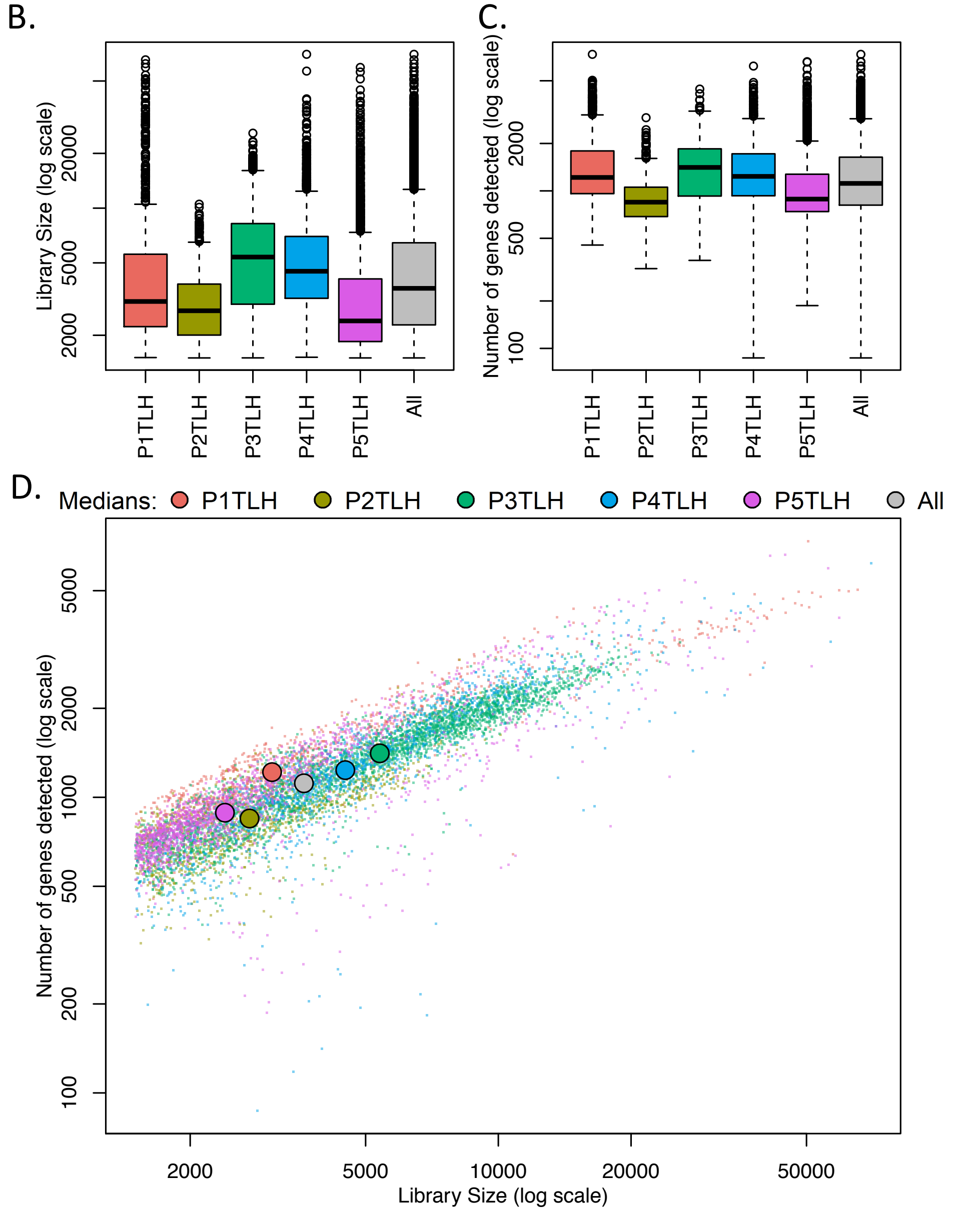

E.

Patient	Mean Library Size (UMIs)	Mean Genes Detected
**P1TLH**	5951	1537
**P2TLH**	3122	906
**P3TLH**	6043	1444
**P4TLH**	6021	1407
**P5TLH**	4164	1149
**All TLHs**	5227	1313

Supplementary Figure 2: Sample quality control. A). From viable cells in the total liver homogenate, as determined by trypan blue exclusion, that were targeted for sequencing, cells were first filtered for library size, then mitochondrial proportion as outlined in the methods. After passing the mitochondrial transcript threshold, all remaining cells per patient were used in downstream analysis. There was an average of 24% recovery rate, with patient 3 as the obvious outlier.
B-E) After removal of cells with more that 50% of transcripts from mitochondria and a library size less than 1500, there was an average library size of 5227 and a mean genes detected per cell of 1313 genes.

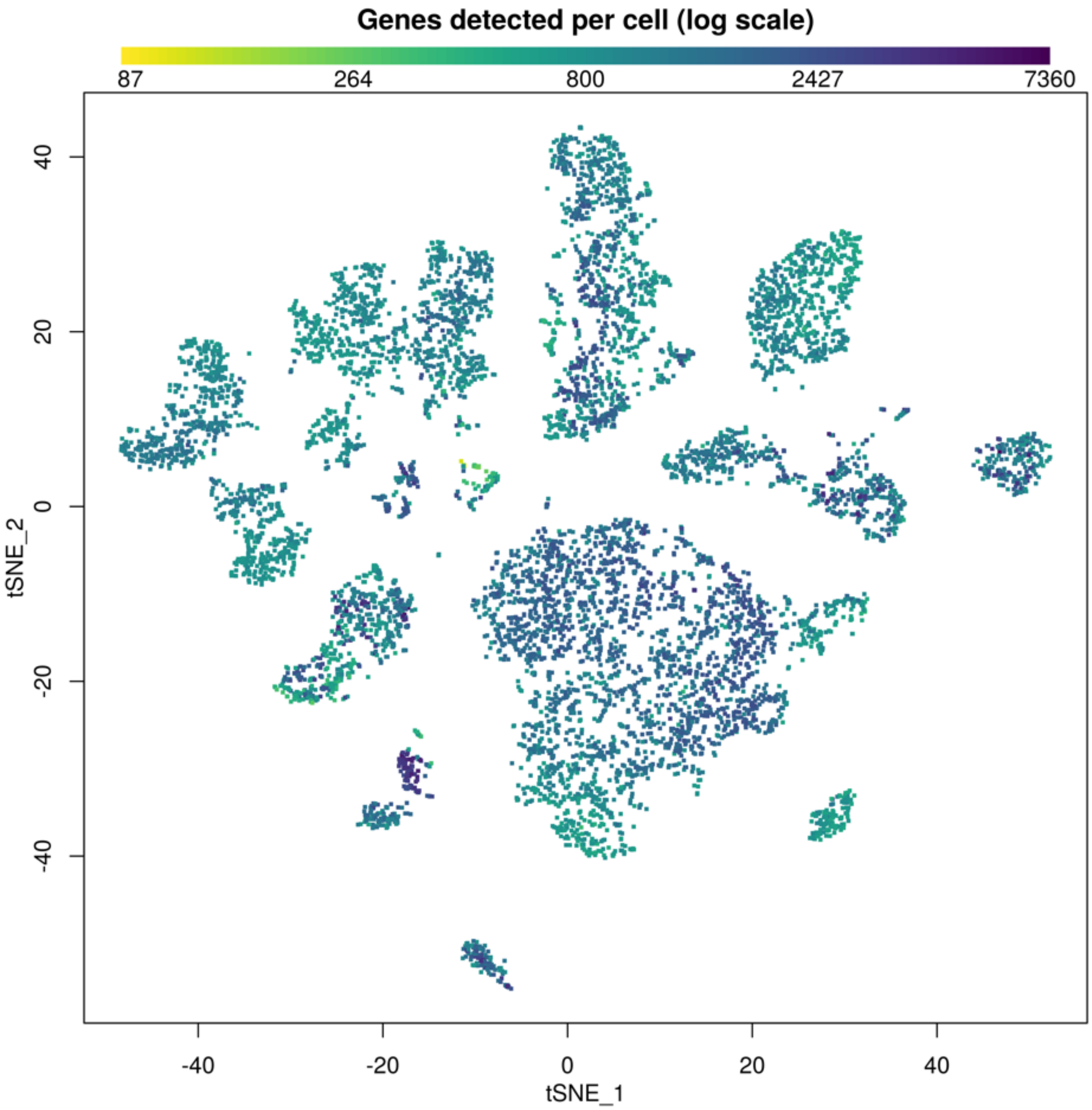

Supplementary Figure 3: Heat map analysis showing the number of genes detected per cell.

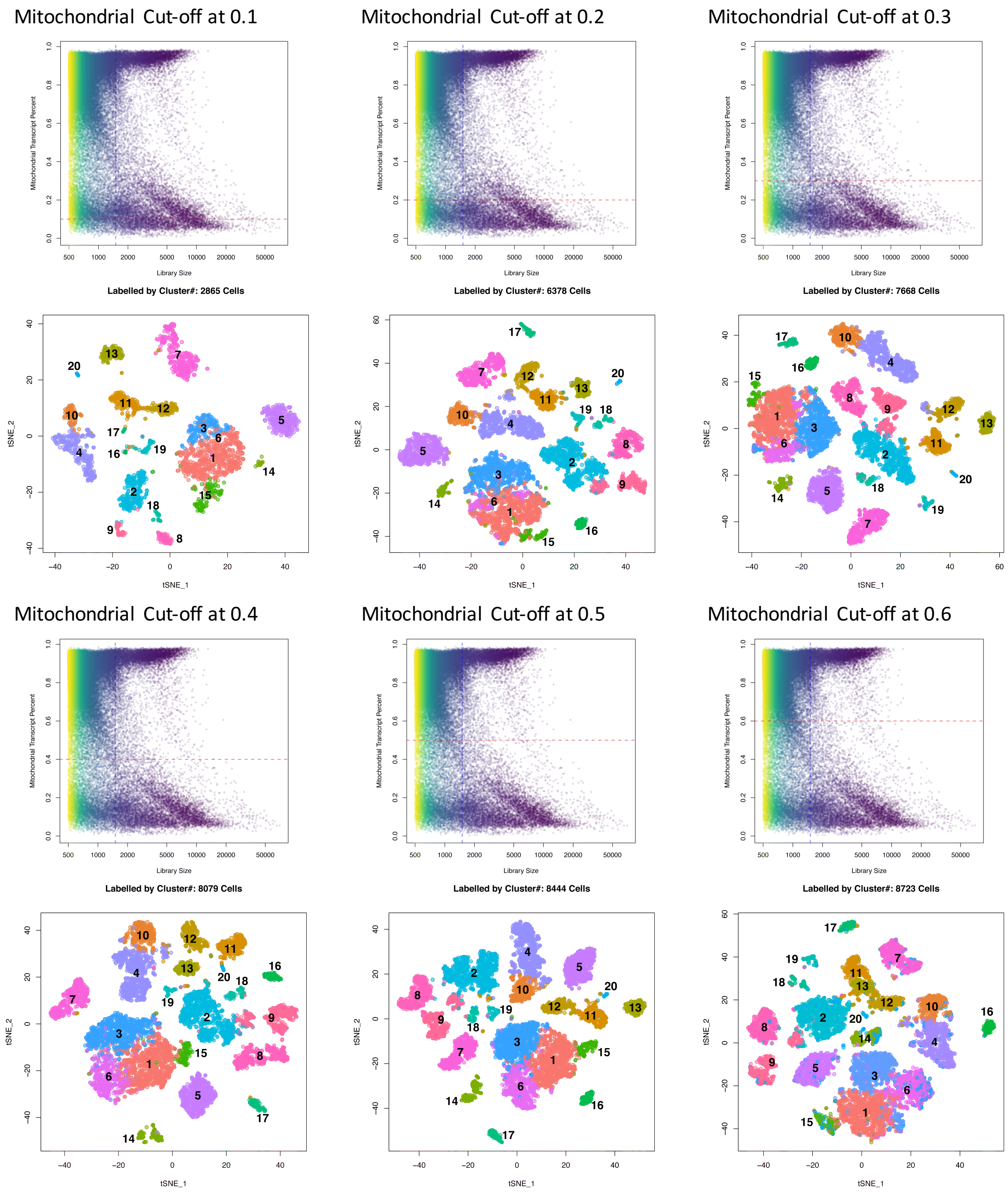

Supplementary Figure 4: Cluster map analysis showing the 6 different mitochondrial transcript cut-offs. In all mitochondrial cut-offs (0.1, 0.2, 0.3, 0.4, 0.5, and 0.6), we have cells from all 20 clusters identified. All clusters (except cluster #6 at 0.1 cutoff) are identified as unique populations in tSNE plot at all cutoffs.

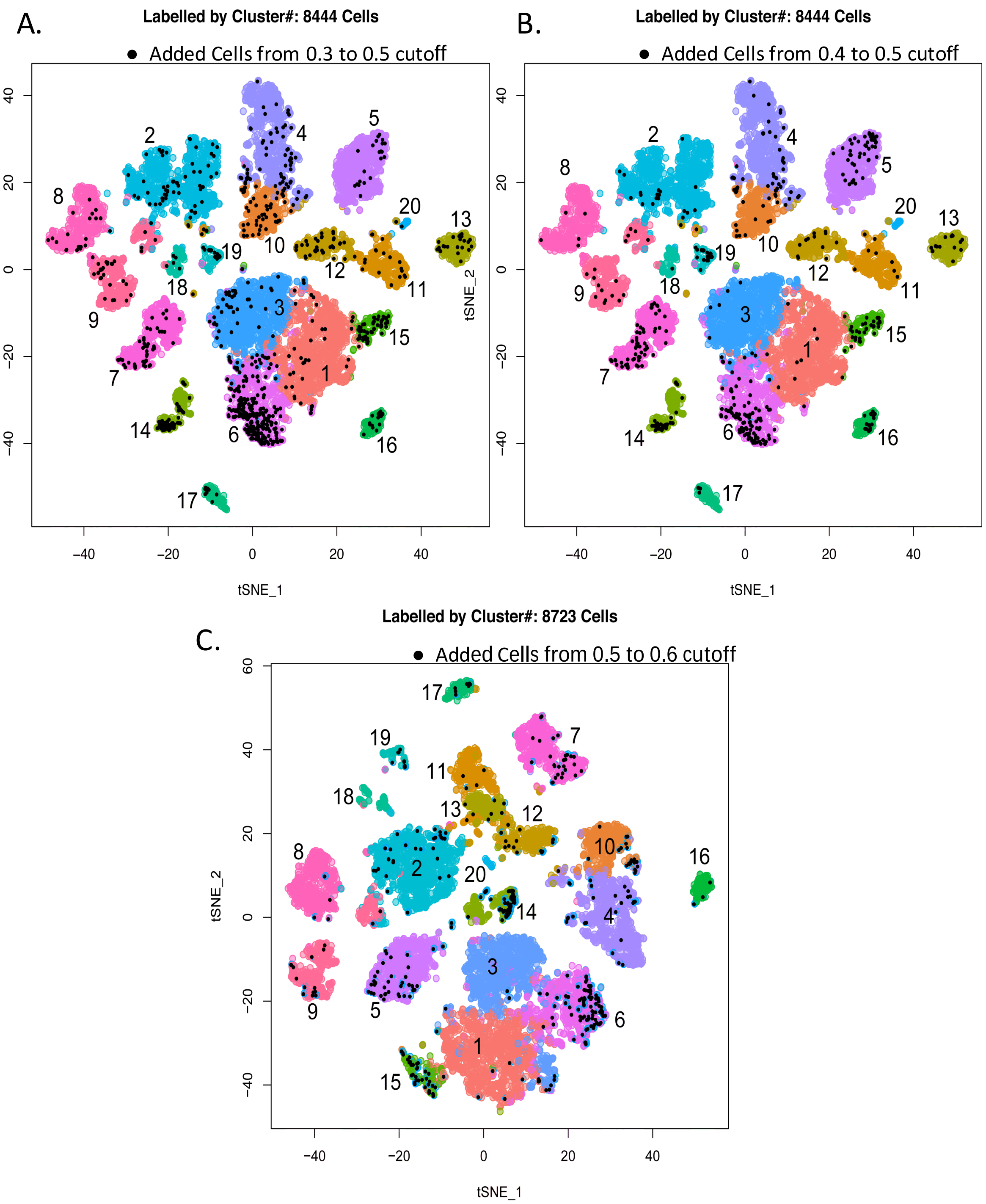

Supplementary Figure 5: Additional cells which appear at various mitochondrial cut-offs. Additional cells from mitochondrial cutoffs: A) 0.3 to 0.5; B) 0.4 to 0.5; and C) 0.5 to 0.6; are found in almost all clusters. This analysis clearly shows that no one cluster or type of cell (hepatocytes) seems biased to include additional cells by changing the mitochondrial transcript threshold. Altogether, our results indicate that the cell clusters identified at 0.5 cutoff is robust and consistent.

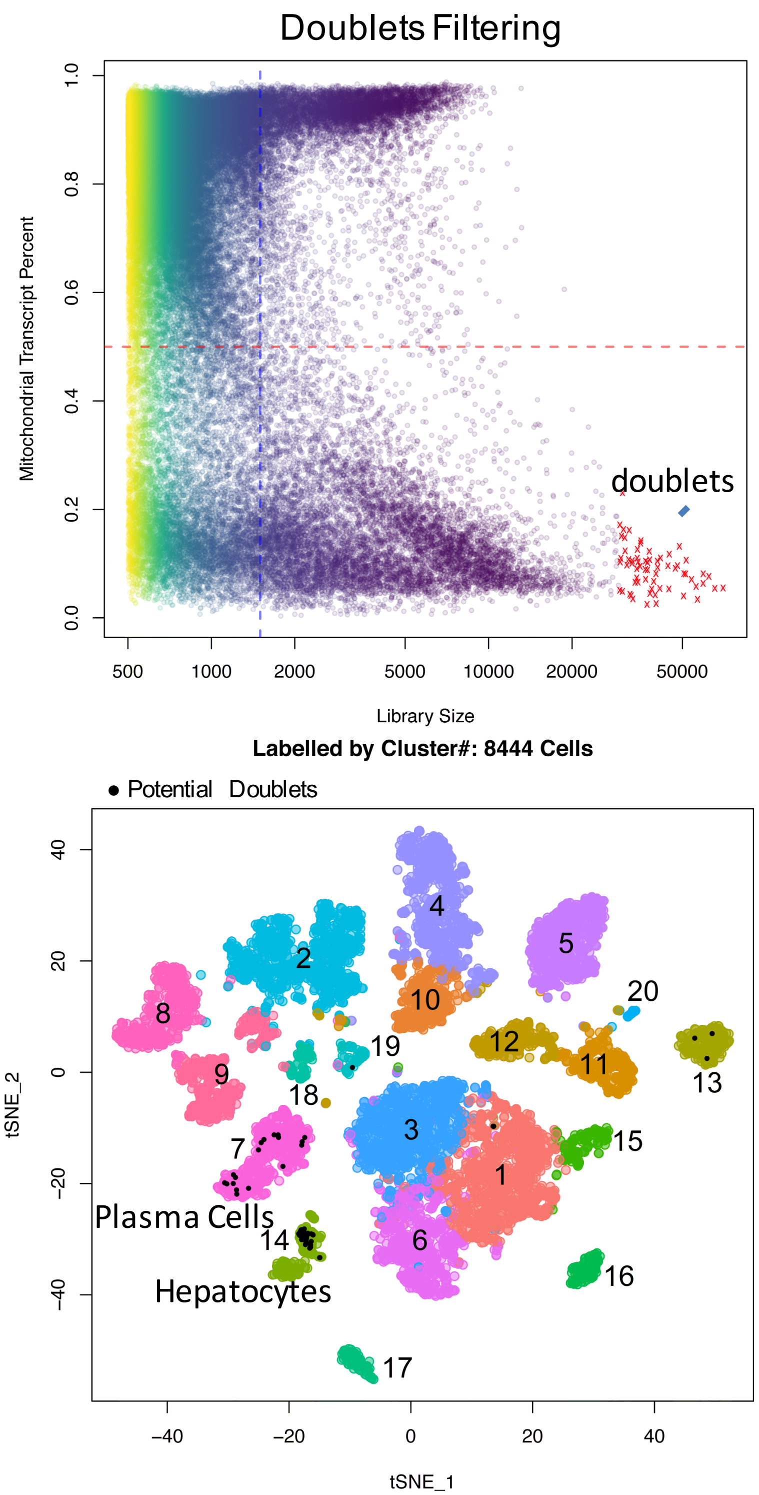

Supplementary Figure 6: Doublet Filtering. We did not apply doublet filtering because there are naturally occurring binucleated hepatocytes in liver and it will be very difficult to distinguish doublets and binucleated cells. Due to the heterogeneity of the liver tissue, it is unlikely that true doublets (with many possible cell type combinations) will result in one unique cluster on tSNE plot. The fact that we see most of the "supposed doublets" are concentrated in the Hepatocyte (cluster #14) and plasma cell (cluster #7) populations indicates these are likely biological cell types.

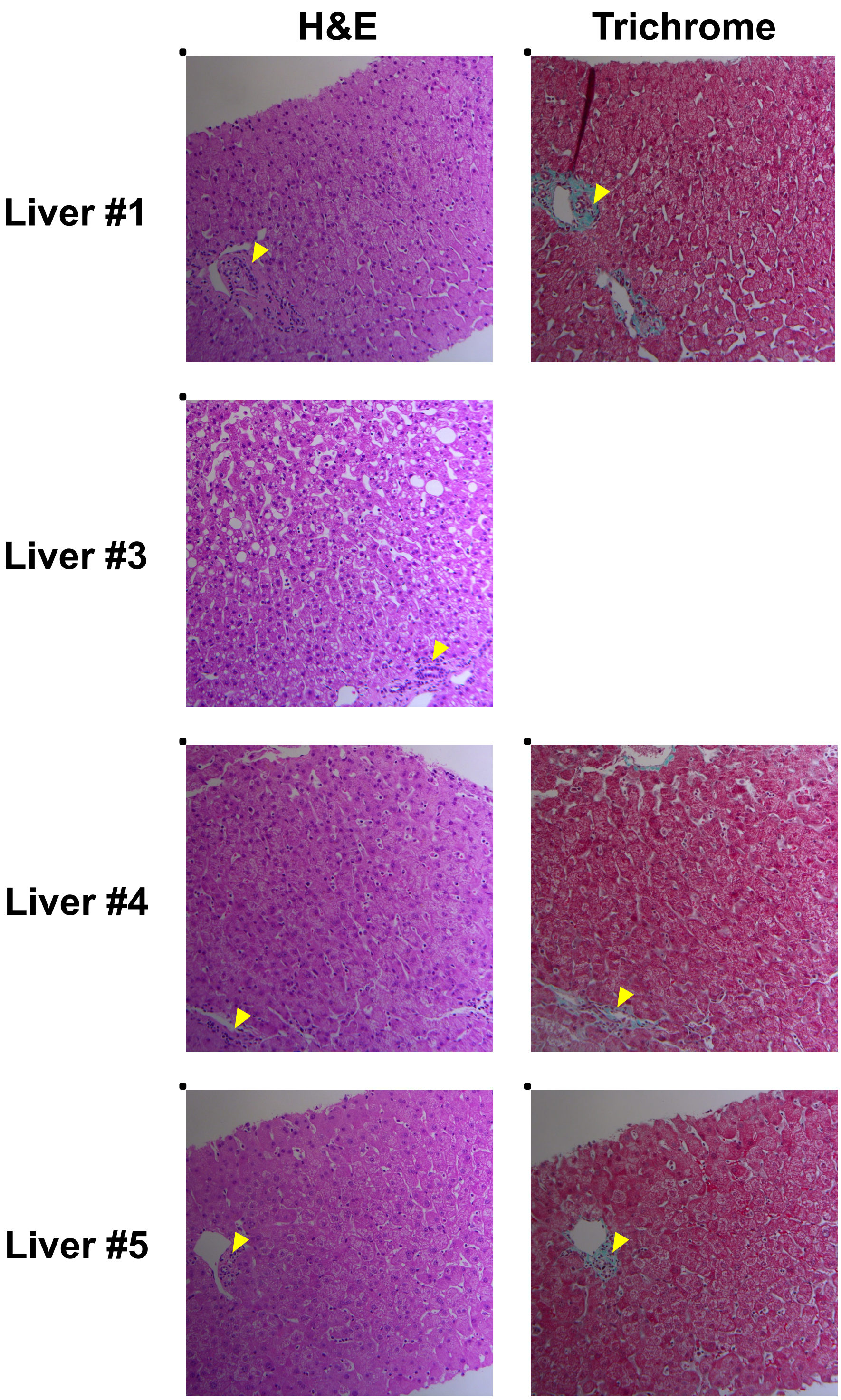

Supplementary Figure 7: Hematoxylin and eosin and Masson's trichrome staining of human liver parenchyma. Livers #1, #4, and #5 show preserved microstructure and absence of steatosis, inflammation, or other abnormality. Liver #3 shows the same normal structure with approximately 5-10% fat, which is acceptable for cadaveric donation. Yellow triangles show portal tracts. Original magnification: 100x. Masson's trichrome staining was not performed on Liver #3.

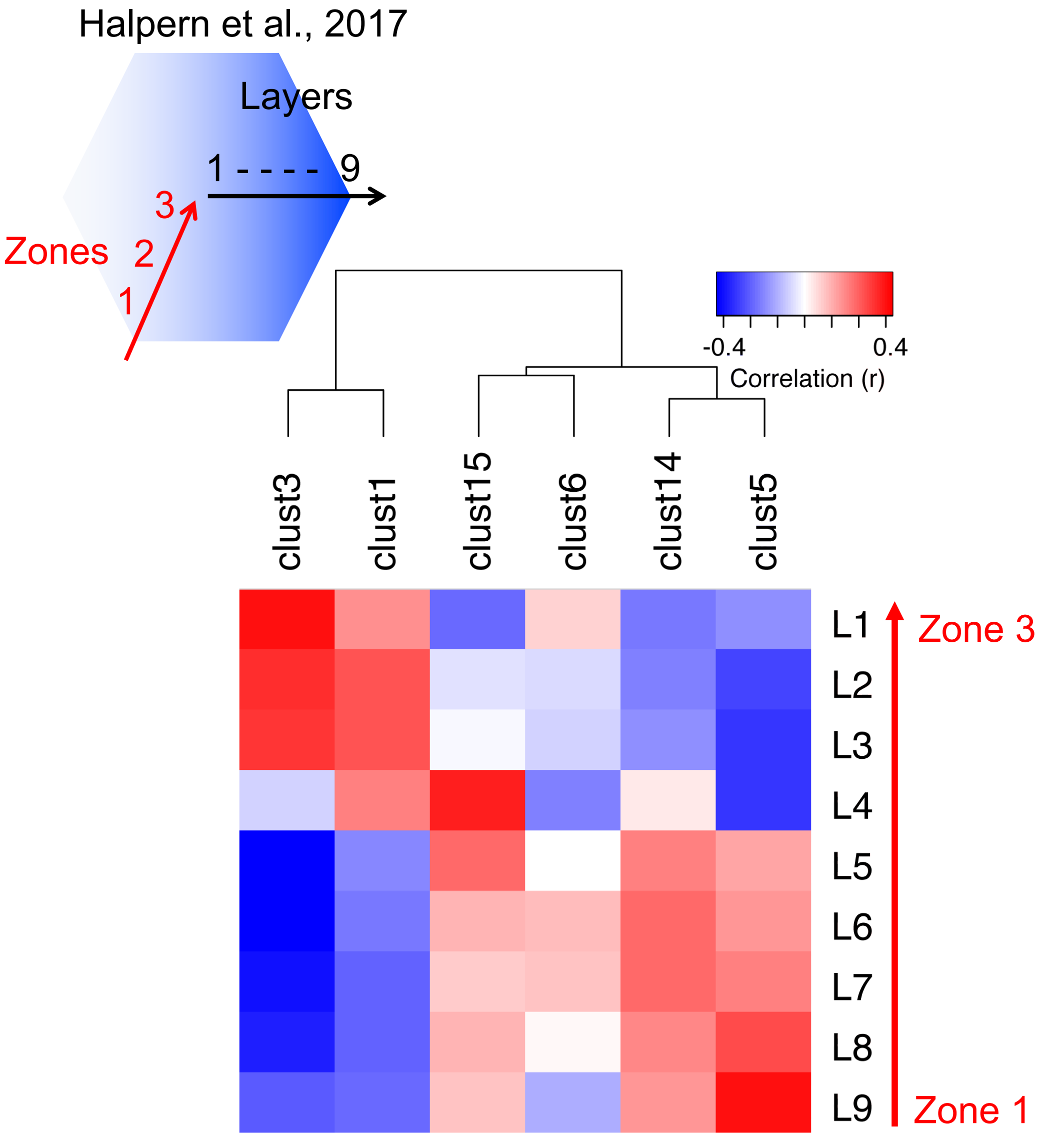

Correlation with highly significant genes (n = 94)
FDR q-values < 1.0 x 10^{-25}

Correlation	Clust3	Clust1	Clust15	Clust6	Clust14	Clust5
L1	0.36***	0.18	-0.16	0.09	-0.15	-0.11
L2	0.31**	0.26*	-0.01	-0.02	-0.14	-0.22*
L3	0.27**	0.24*	0.01	-0.03	-0.12	-0.23*
L4	-0.05	0.11	0.21*	-0.12	0.01	-0.20
L5	-0.44***	-0.22*	0.19	-0.04	0.16	0.10
L6	-0.38***	-0.21*	0.07	0.06	0.17	0.11
L7	-0.31**	-0.21*	0.04	0.04	0.15	0.13
L8	-0.30**	-0.22*	0.06	-0.02	0.12	0.19
L9	-0.21	-0.19	0.04	-0.12	0.09	0.24*

***P<0.001, **P<0.01, *P<0.05.

Supplementary Figure 8: Similarity of human hepatocyte clusters to known mouse liver sinusoid layers. Homologous genes between mouse and human were first identified using Ensembl databases by the getLDS function in the BioConductor package BioMart. Using the average values of nine layers of mouse liver cells provided in the Halpern study (Halpern et al.., Nature, 2017), highly significant genes (q-values < 1.0 x 10E-25, n = 94) were selected for correlation. Expression values of each gene among the six clusters of human hepatocytes and nine layers of mouse liver cells were then scaled and centered (separately in human and mouse) by z-scores. Finally, Pearson correlation was calculated using z-scores to compare the six human hepatocytes clusters with nine layers of mouse liver cells.

Hepatocyte Markers

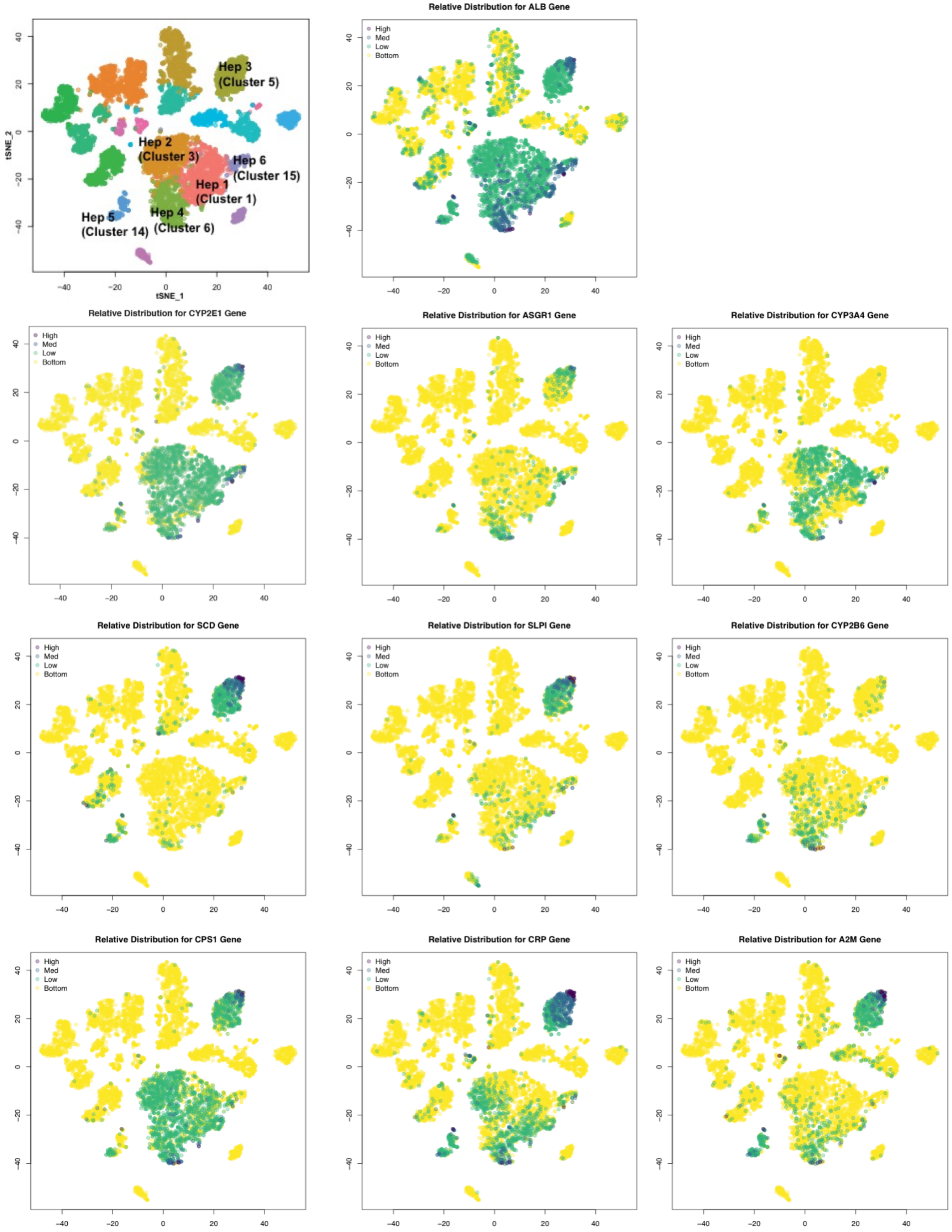

Supplementary Figure 9: t-SNE plots showing the relative distribution of commonly expressed hepatocyte genes in the healthy liver. Legend for relative expression of each marker from lowest expression (yellow dots) to highest expression (Purple dots) (top left).

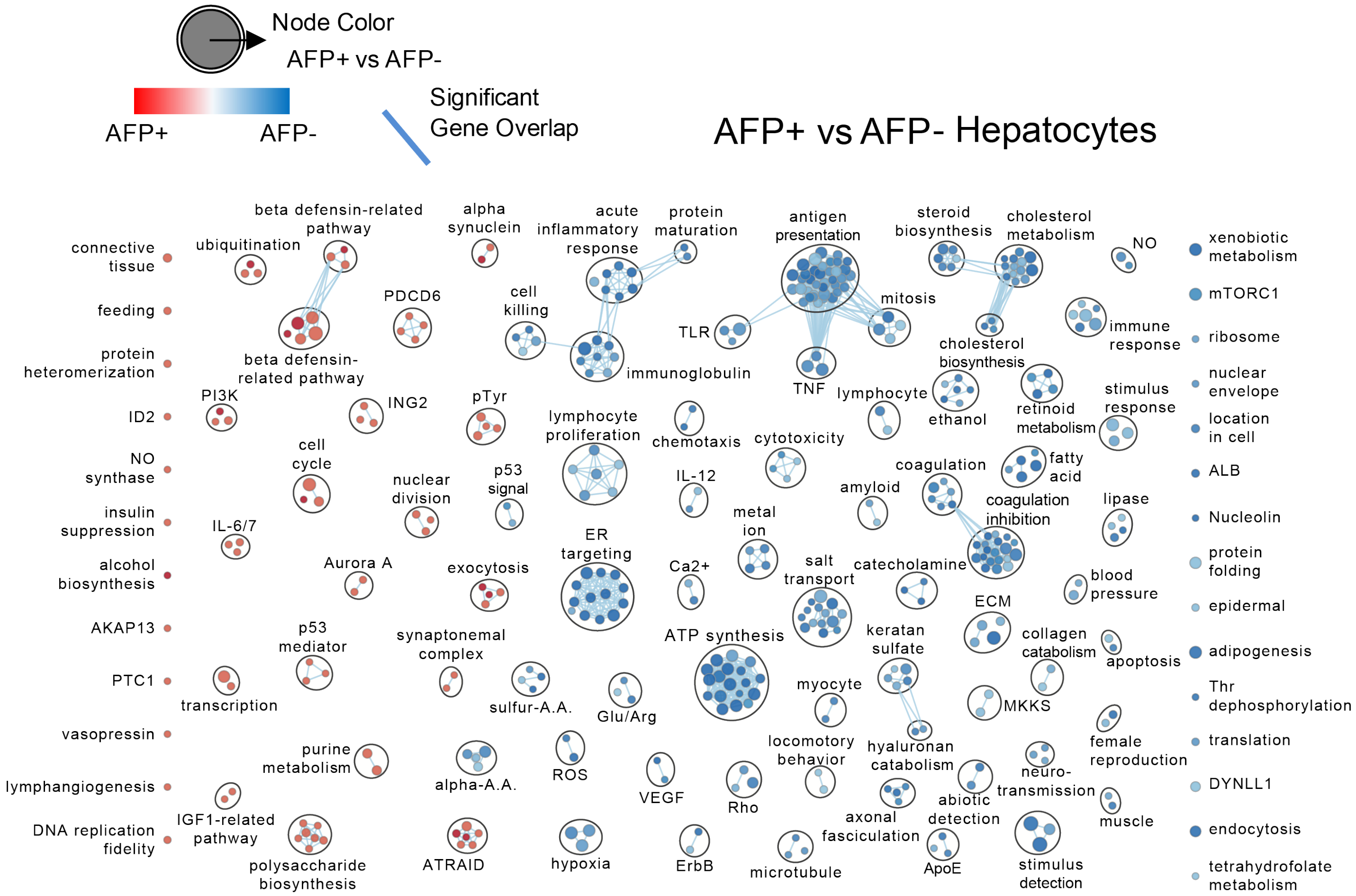

Supplementary Figure 10: Pair-wise analysis of AFP+ and AFP- cells in all hepatocyte clusters (Clusters 1, 3, 5, 6, 14, 15). Pair-wise pathway enrichment analysis using GSEA software on AFP^- cells in clusters 1, 3, 5, 6, 14 & 15 defined in **Fig 2f** compared to AFP^+ cells (shown in Fig. **4c.v.**). Pathways enriched in AFP^+ cells are labeled in red and pathways enriched AFP^- cells are indicated in blue. The size of the node represents the number of genes in a particular pathway (black circle). Blue lines depict intra- and inter-pathway relationships according to the number of genes shared between each pathway. Log2 CPM cut-off to separate AFP^+ (Low/Medium and High expression) and AFP^- (Bottom expression) was 1.668 CPM. This cut-off was determined automatically by binning in R (cut function). The data shows that active cellular pathways in AFP^- are typical of mature hepatocytes while AFP^+ cells were enriched for cellular pathways including cell cycle, nuclear division, IL-6/7 suggesting that these may be hepatic progenitors.

Endothelial Cell Markers

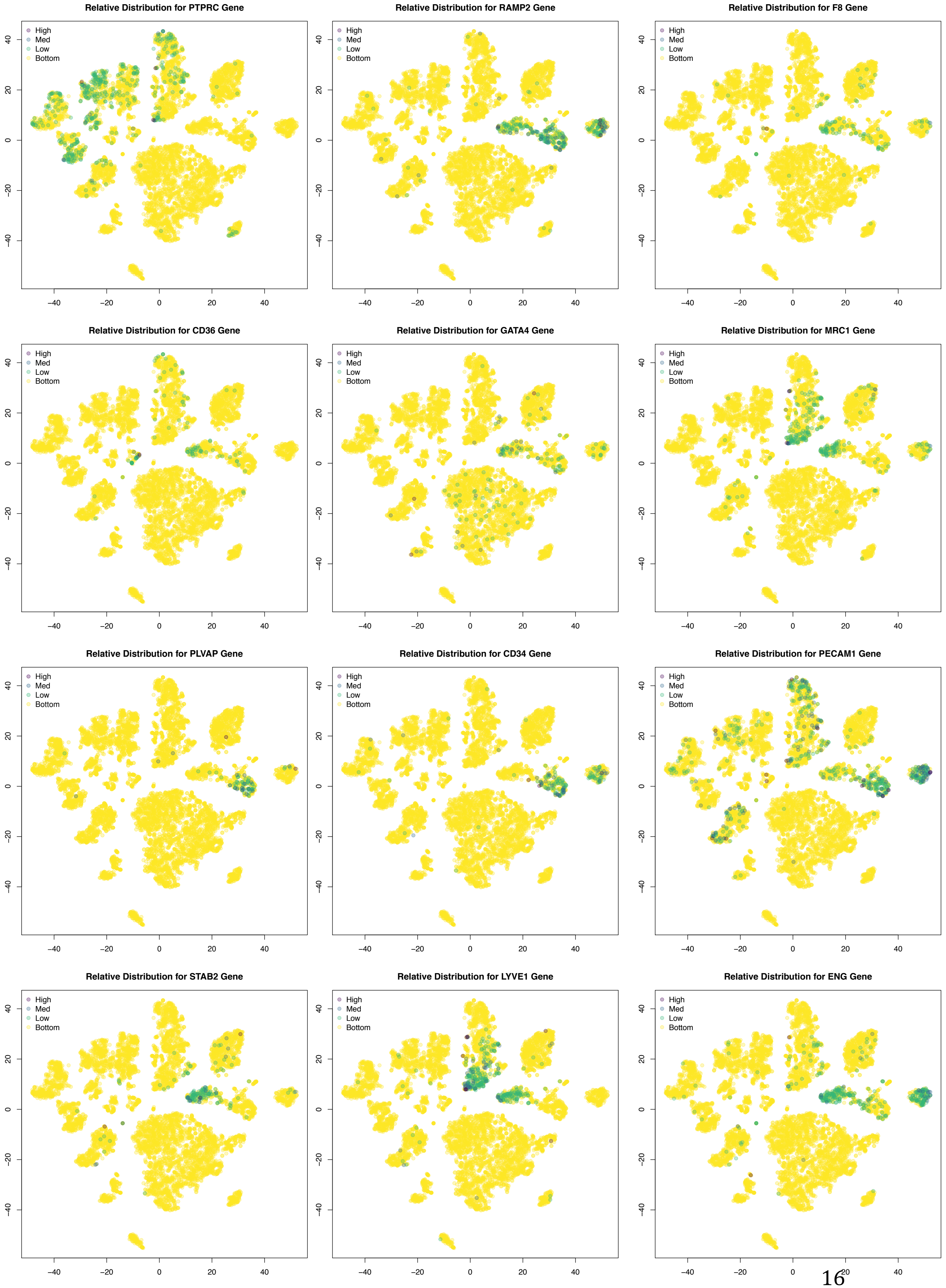

Supplementary Figure 11: t-SNE plots showing the relative distribution of commonly expressed endothelial cell genes in the healthy liver. Legend for relative expression of each marker from lowest expression (yellow dots) to highest expression (purple dots) (top left).

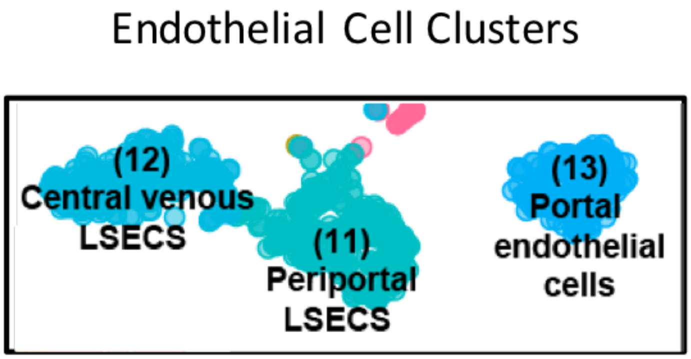

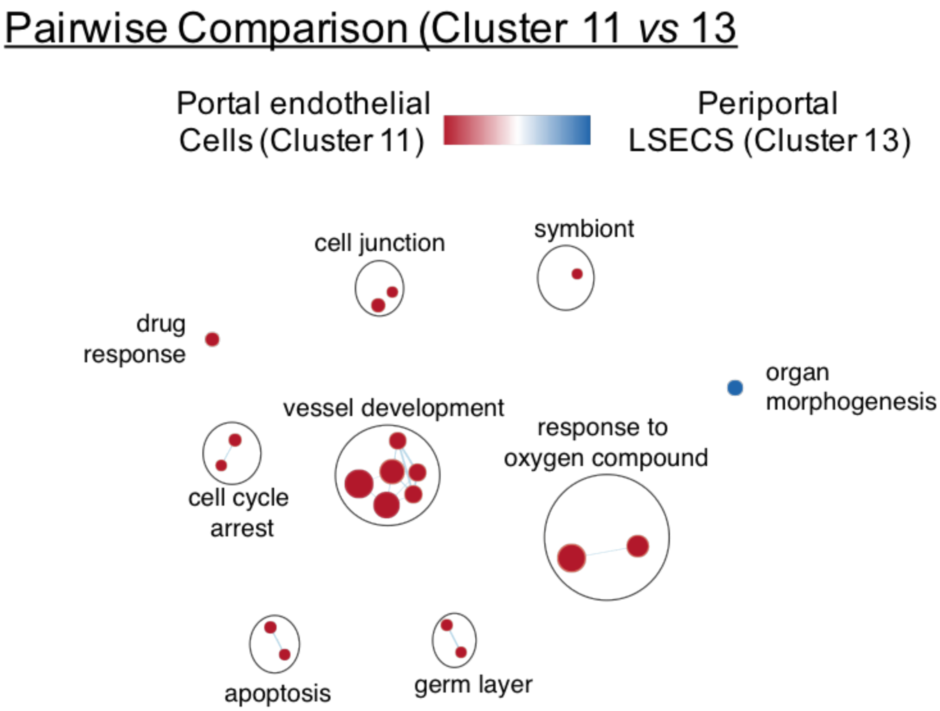

Supplementary Figure 12: Pair-wise pathway analysis of liver endothelial cell populations 11 and 13 reveal few unique pathways activated. Pair-wise pathway enrichment analysis using GSEA software on the clusters 11 and 13 defined in Fig 2f. Pathways enriched (pairwise analysis explained in the legend to Figure 5d) in periportal LSECs are labelled in blue and pathways enriched in portal endothelial cells are indicated in red. The size of the node represents the number of genes in a particular pathway (black circle). Blue lines depict intra- and inter-pathway relationships according to the number of genes shared between each pathway.

Endothelial Cell Clusters

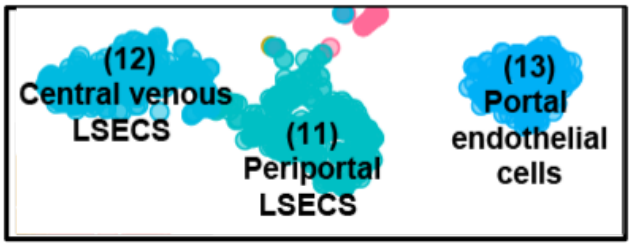

Pairwise Comparison (Cluster 13 *vs* 12)

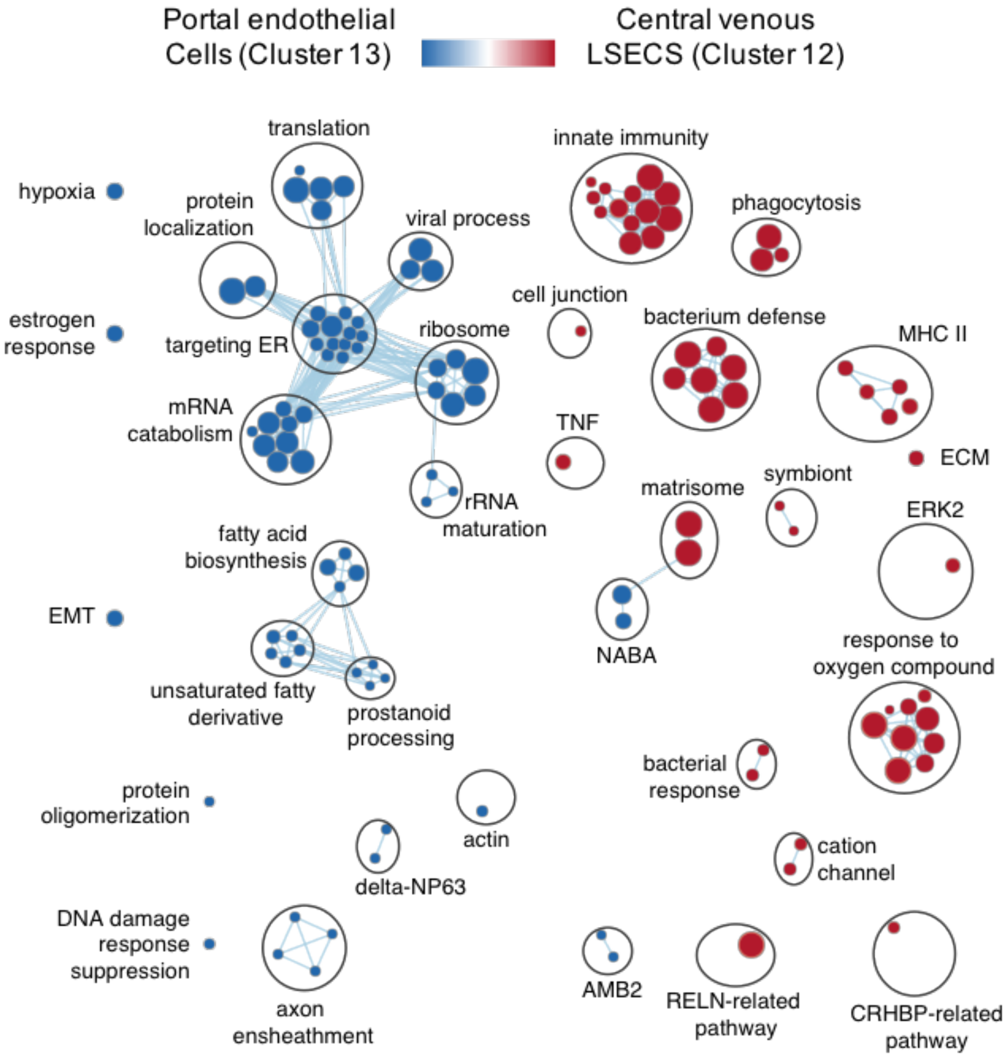

Supplementary Figure 13: Pair-wise pathway analysis of liver endothelial cell populations 12 and 13 reveal unique pathways activated. Pair-wise pathway enrichment analysis using GSEA software on the clusters 12 and 13 defined in Fig 2. Pathways enriched in portal endothelial cells are labelled in blue and pathways enriched in central venous LSECs are indicated in red. The size of the node represents the number of genes in a particular pathway (black circle). Blue lines depict intra- and inter-pathway relationships according to the number of genes shared between each pathway.

Kupffer Cell/ Macrophage Markers

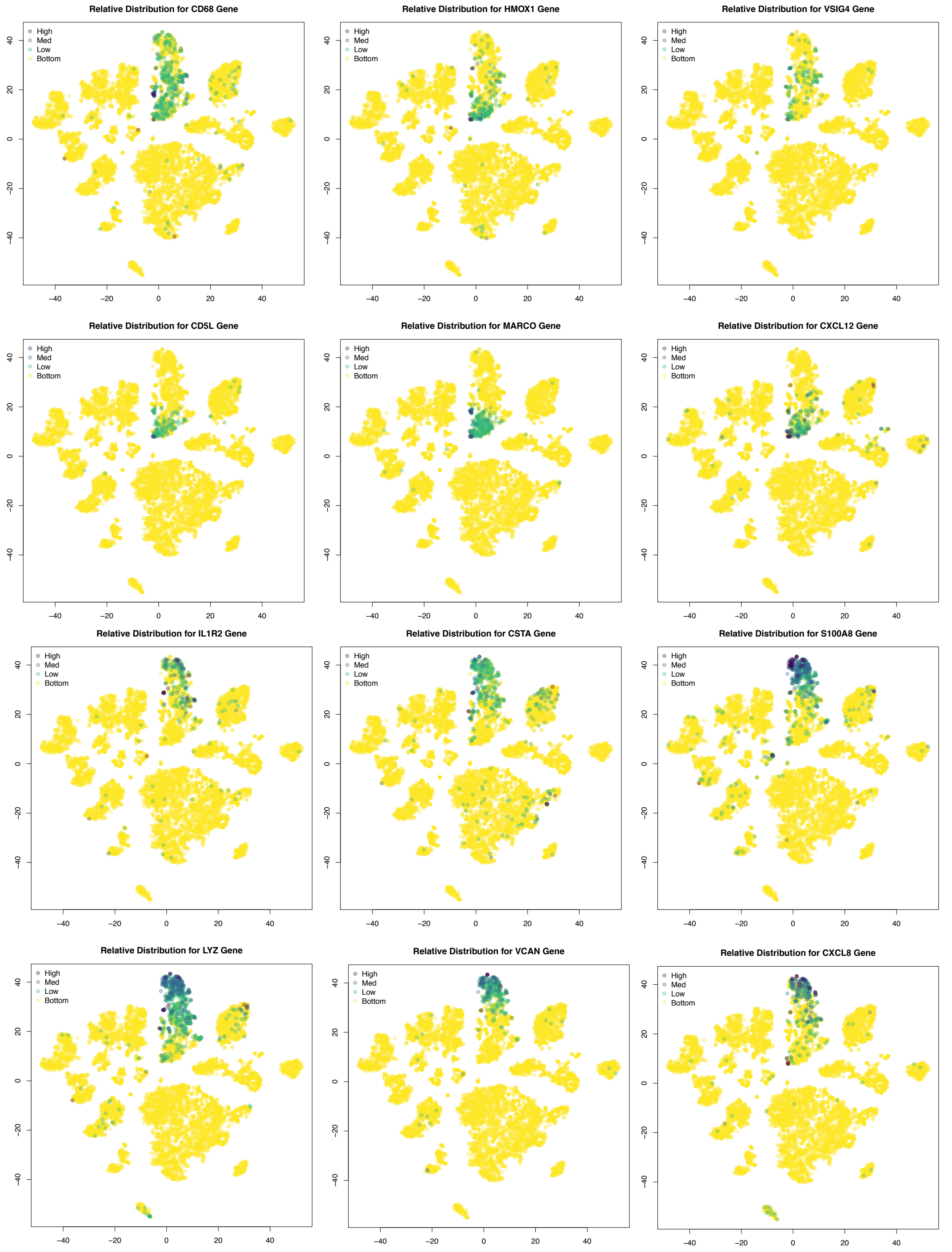

Supplementary Figure 14: t-SNE plots showing the relative distribution of commonly expressed macrophage genes in the healthy liver. Legend for relative expression of each marker from lowest expression (yellow dots) to highest expression (purple dots) (top left).

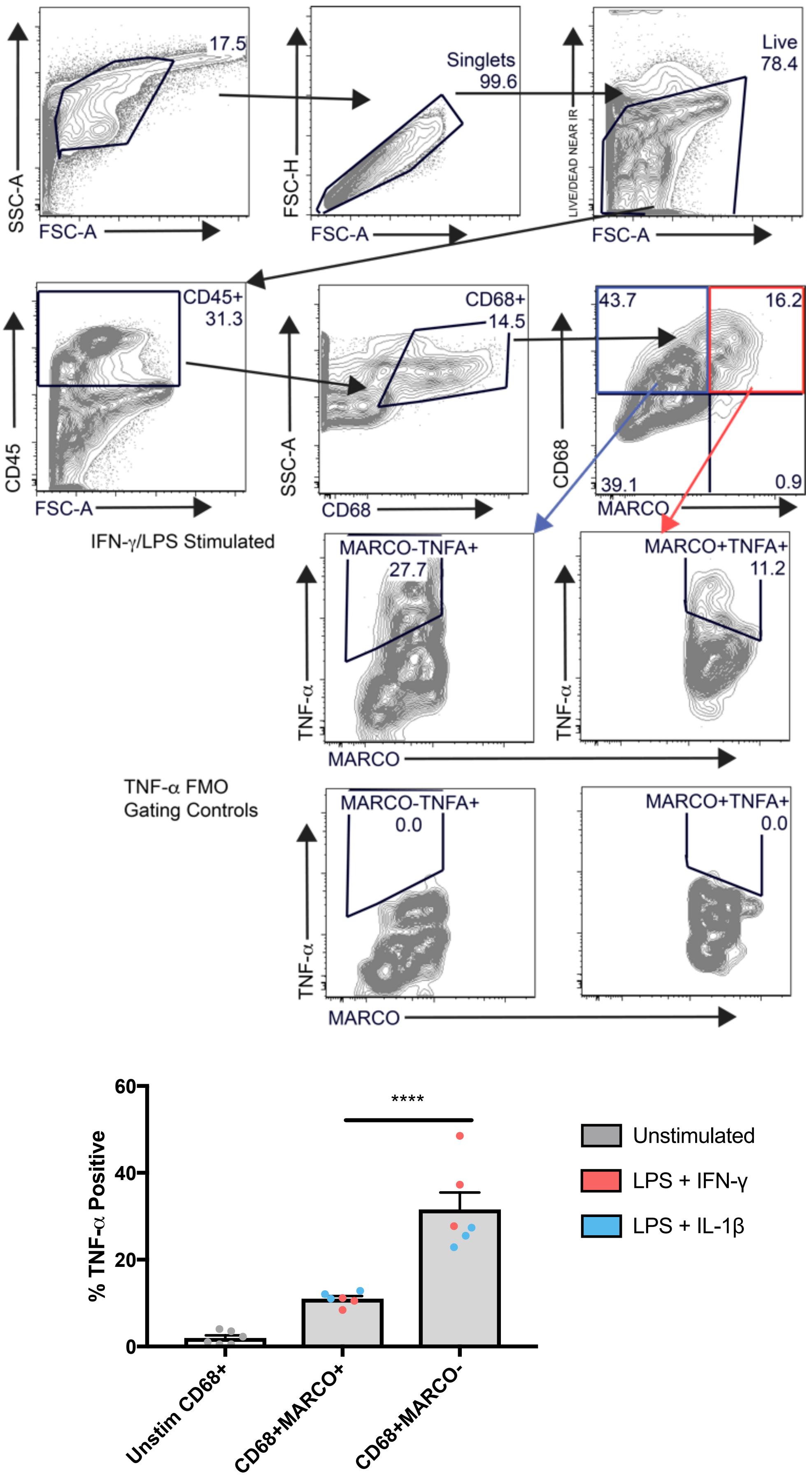
SSC-A
FSC-A
17.5
FSC-H
Singlets
99.6
LIVE/DEAD NEAR IR
Live
78.4
CD45
CD45+
31.3
SSC-A
CD68
CD68+
14.5
43.7
16.2
39.1
0.9
MARCO
IFN-γ/LPS Stimulated
MARCO-TNFA+
27.7
MARCO+TNFA+
11.2
TNF-α
MARCO
TNF-α FMO
Gating Controls
MARCO-TNFA+
0.0
MARCO+TNFA+
0.0
% TNF-α Positive
60
40
20
0
****
Unstim CD68+
CD68+MARCO+
CD68+MARCO-
Unstimulated
LPS + IFN-γ
LPS + IL-1β

Supplementary Figure 15: Gating Strategy for Fig. 8d. Cell suspensions from TLH were stained with a live/dead aqua dye to label dead cells, fluorophore-conjugated monoclonal antibodies to human cell-surface markers anti-CD45-BV650 (Biolegend Clone: HI30), anti-CD68-PE (Biolegend Clone: Y1/82A), and anti-MARCO (rabbit anti-human polyclonal) (Thermofisher: PA5-26888); secondary donkey anti-rabbit-FITC (Invitrogen). Singlets were defined as having similar area and height measurements in forward scatter (FSC-A vs FSC-H). Gating strategy for cell surface markers was set based on background auto-fluorescence measured in unstained controls. MARCO staining was used to differentiate between the two predominant macrophage populations found in the liver. Gating for TNF-α secretion was set based on fluorescence-minus-one (FMO) controls. Error bars show standard error of the mean for 6 replicates. Statistical significance evaluated using a one-way analysis of variance (ANOVA) with a Bonferroni post-test ****$P<0.0001$.

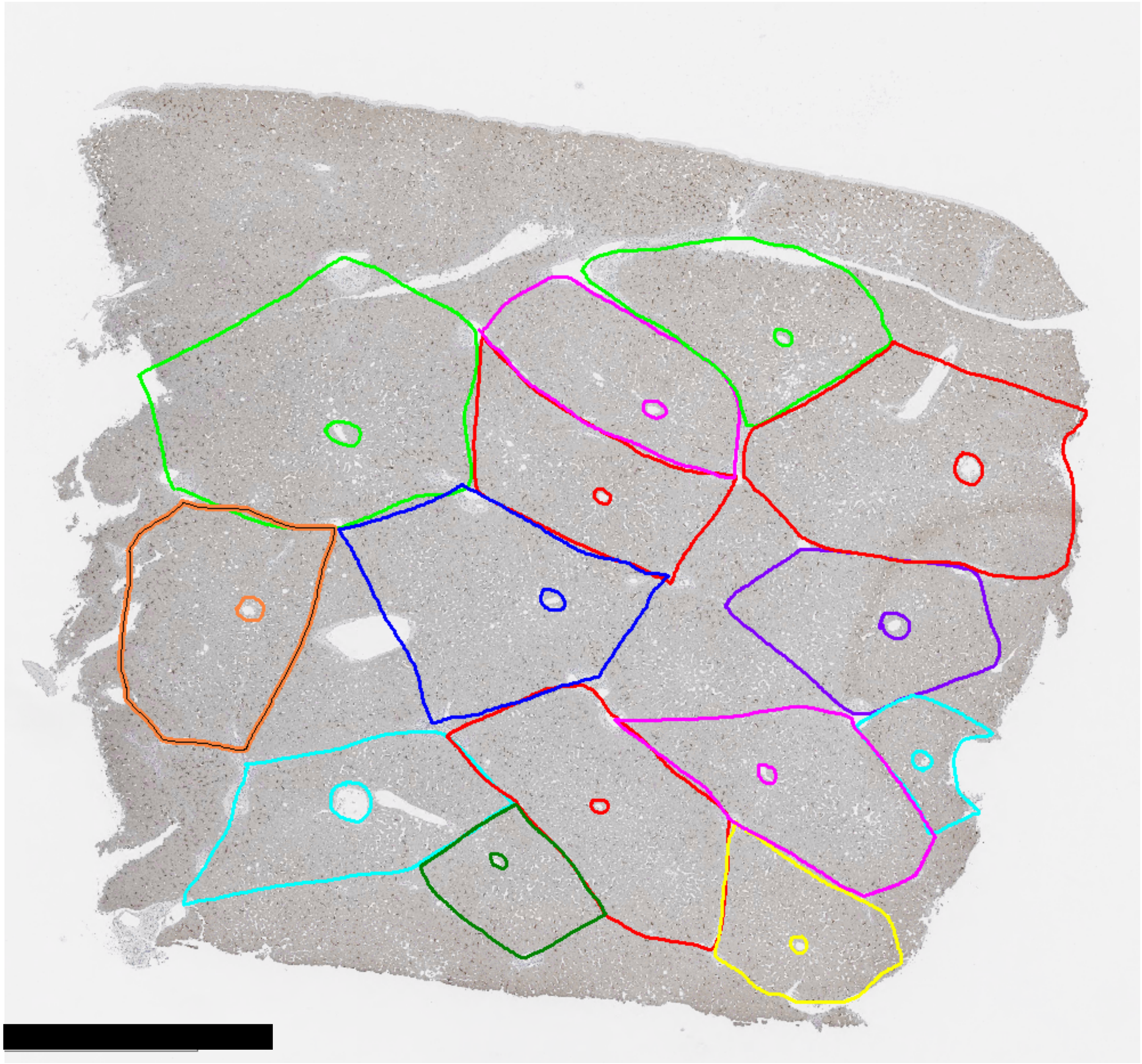

Supplementary Figure 16: Representative partitioning of lobules for immunohistochemistry (Figure 8 e-f). Lobules were defined by drawing a continuous line between portal triads around a single central vein using Halo software (Indica Labs, version). Each lobule was then concentrically partitioned into 10 layers between outer portal vein (layer 1) towards the central vein (layer 10). The positively stained area within each layer of the lobule (10 layers/lobule) was quantified and normalized to the area of the layer and presented as % positive stain. Scale bar indicates 2mm.

CD68 40x mag

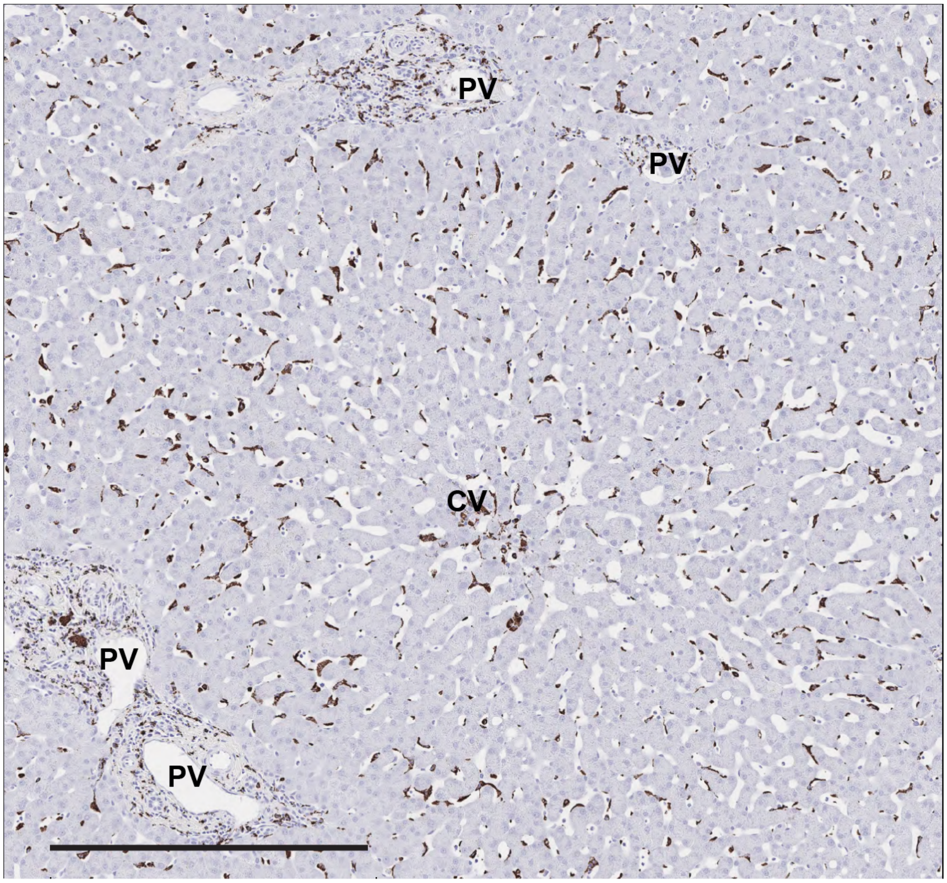

MARCO 40x mag

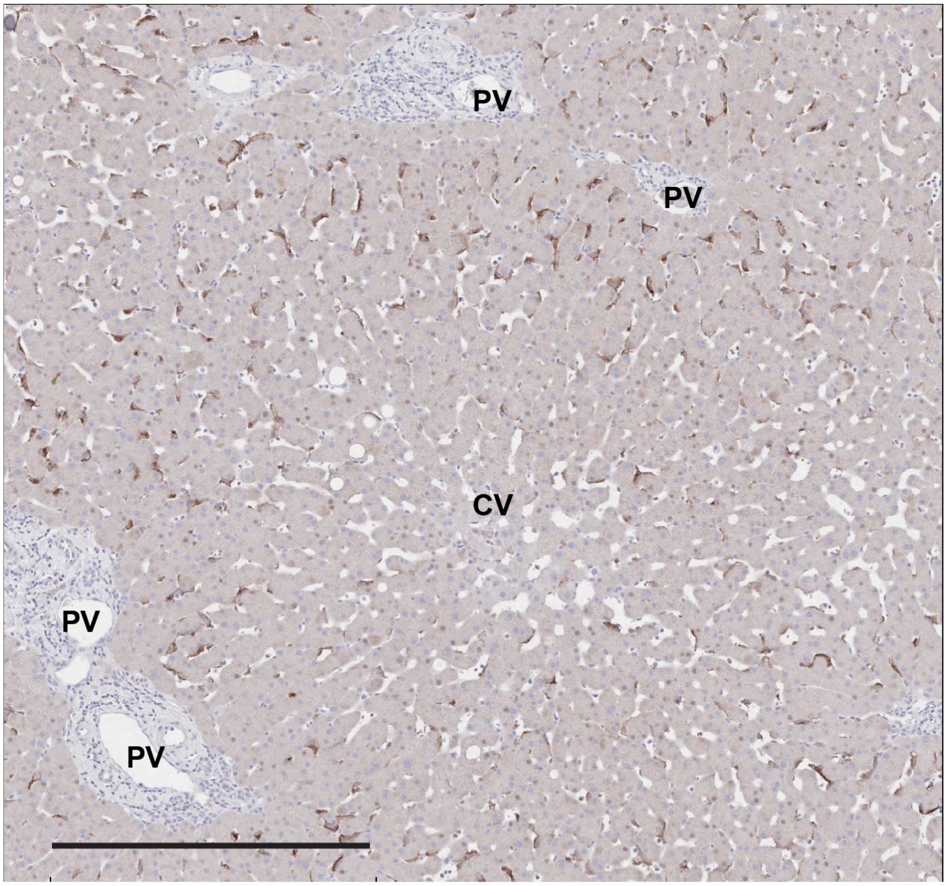

Supplementary Figure 17: Distribution of macrophages by CD68 (general macrophage marker) vs. MARCO (non-inflammatory KCs). Human liver tissue resected from neurologically deceased donors was cut into 4mm x 4mm x 4mm blocks and fixed in 10% formalin. resected from neurologically brain-dead donors. MARCO (Invitrogen, PA5-26888, 1:300) and CD68 (DAKO, PG-M1, 1:600) staining was performed on sequentially cut 7 μm slides cut from paraffin-embedded liver tissue by the Toronto Pathology Research Program (Toronto General Hospital) using standard methods. Staining was performed with LT TE9 treated slides; primary antibodies were detected using a donkey anti-mouse or -human secondary antibody conjugated to HRP. Scale bar indicates 500μm.

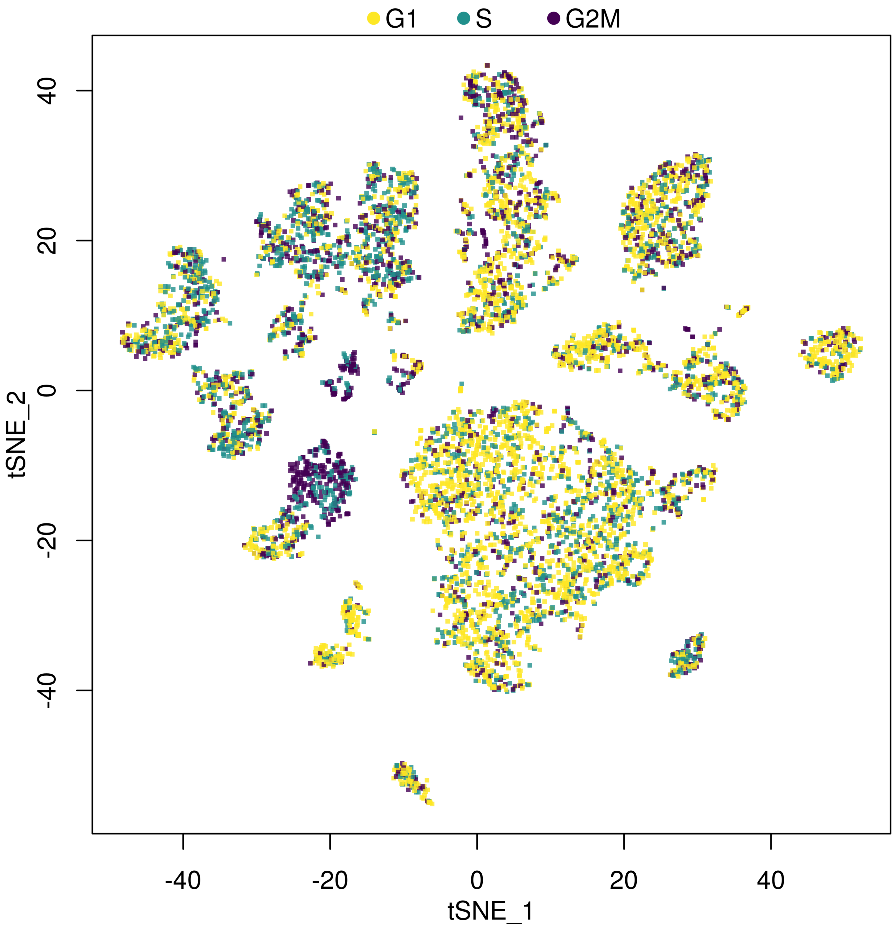

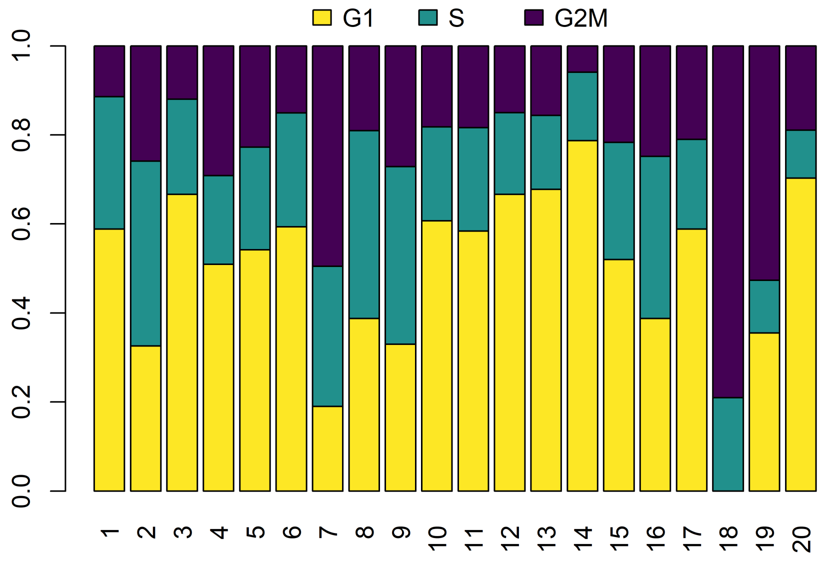

Supplementary Figure 18: Cell cycle phase in each cluster. Identification of cell cycle stage for 8444 cells profiled. G1-yellow, S-aqua, G2/M-purple. Cell cycle phases were predicted using the CellCycleScoring function in the Seurat R package.

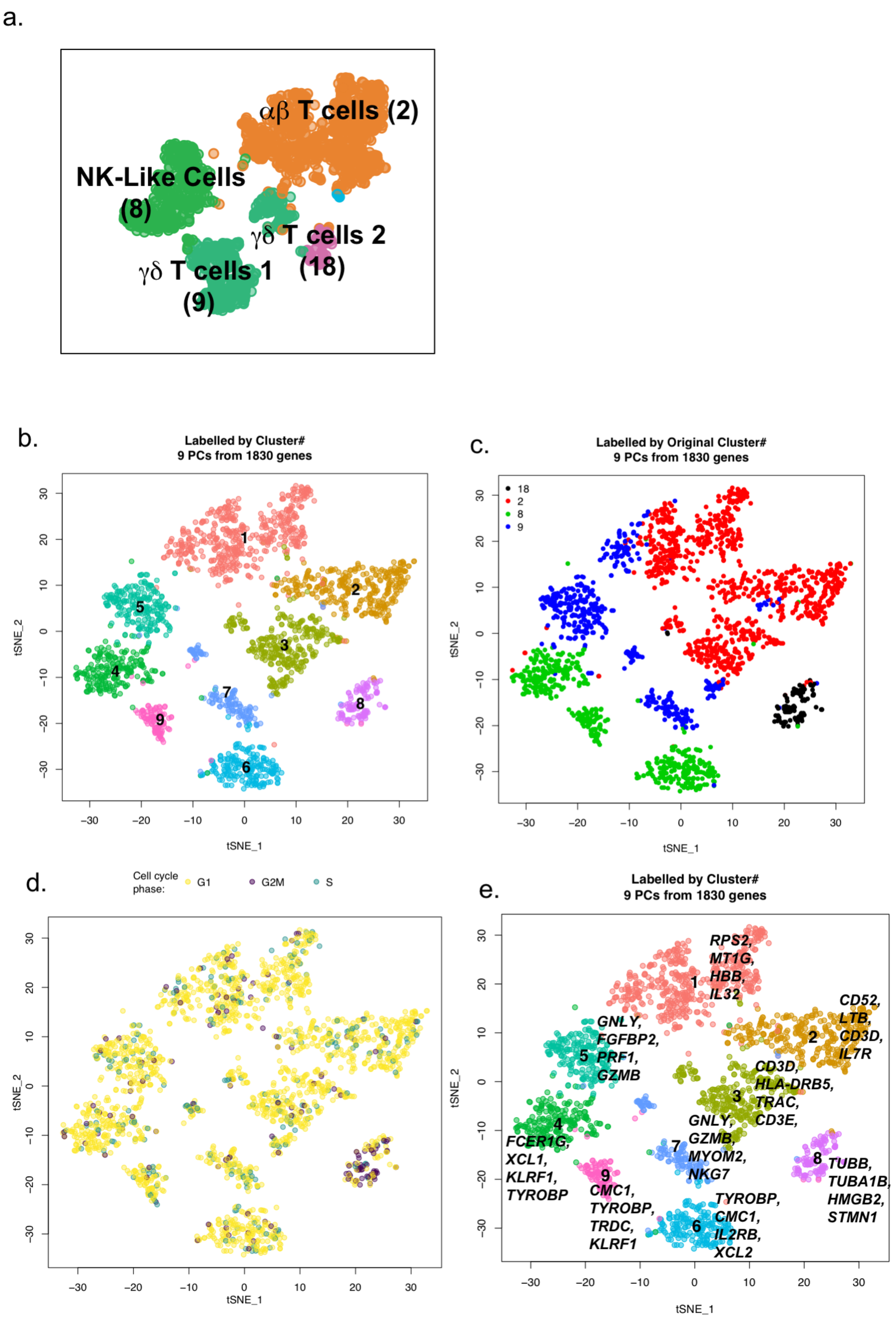
a.
αβ T cells (2)
NK-Like Cells (8)
γδ T cells 2 (18)
γδ T cells 1 (9)
b.
Labelled by Cluster#
9 PCs from 1830 genes
tSNE_1
tSNE_2
c.
Labelled by Original Cluster#
9 PCs from 1830 genes
18
2
8
9
tSNE_1
tSNE_2
d.
Cell cycle phase:
G1
G2M
S
tSNE_1
tSNE_2
e.
Labelled by Cluster#
9 PCs from 1830 genes
RPS2, MT1G, HBB, IL32
CD52, LTB, CD3D, IL7R
GNLY, FGFBP2, PRF1, GZMB
CD3D, HLA-DRB5, TRAC, CD3E,
GNLY, GZMB, MYOM2, NKG7
FCER1G, XCL1, KLRF1, TYROBP
CMC1, TYROBP, TRDC, KLRF1
TYROBP, CMC1, IL2RB, XCL2
TUBB, TUBA1B, HMGB2, STMN1
tSNE_1
tSNE_2

Supplementary Figure 19: T and NK-like cell sub-analysis reveals additional distinct populations. Potential conventional and non-conventional T cell and NK-like cell populations in (a-b) Clusters 2, 8, 9 and 18 were identified by rerunning dimensionality reduction and clustering analysis. (c) Newly identified clusters labelled by original cluster number. (d) Transcriptome-based heat map representation of cell cycle status (G1, G2/M, or S) in every cell within a cluster (as defined in (b)). (e) Cluster map of cell subpopulations with the top 4 differentially expressed genes indicated (ranked by p-value). Full Seurat Parameters: FindClusters(Data,pc.use=1:9,print.output=F,save.SNN=T,resolution=0.4)
DE: differentially expressed; PCA: principal component analysis; *t*-SNE: t-distributed stochastic neighbor embedding; PCs: (Principal Components).

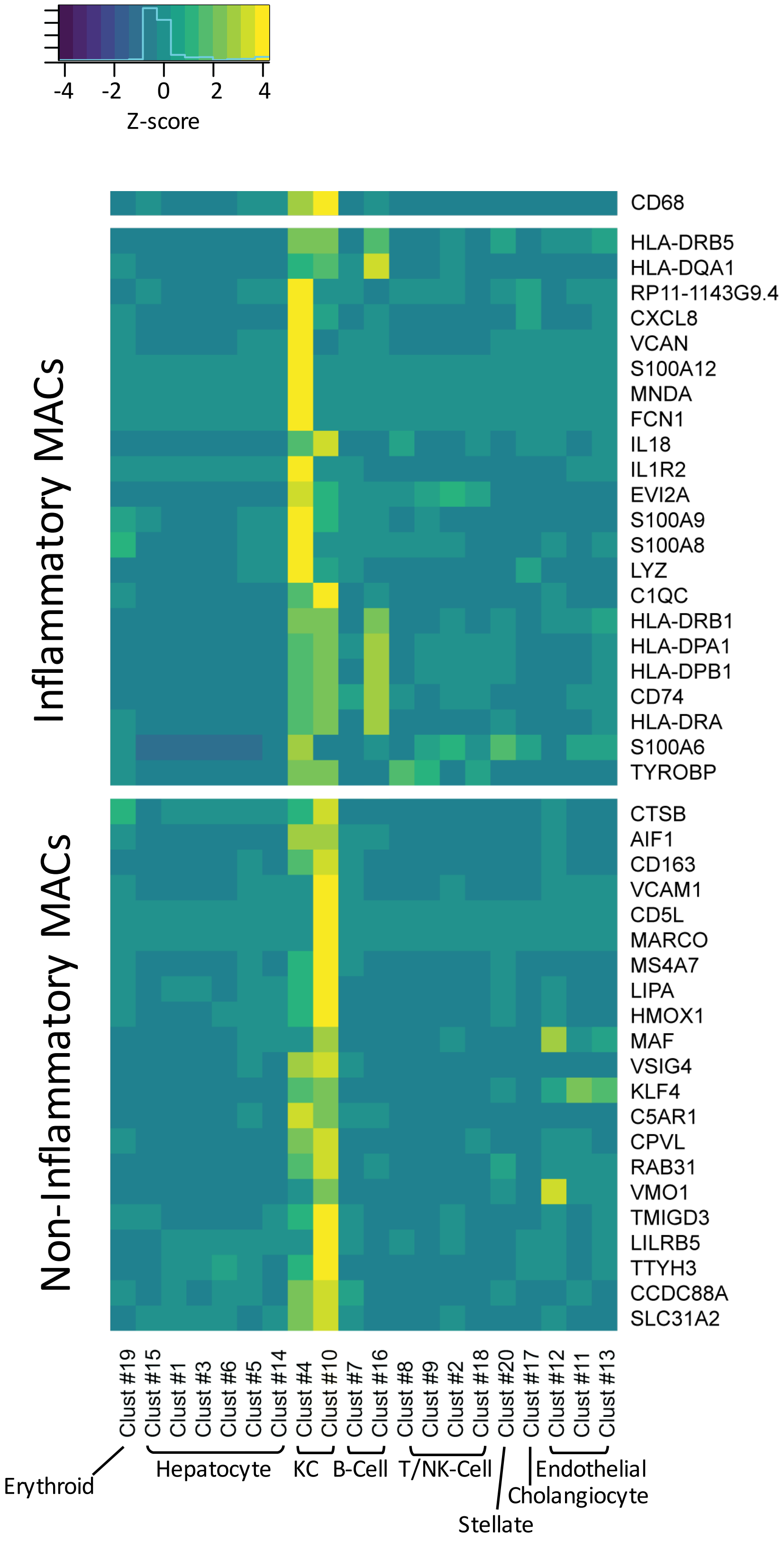

Supplementary Figure 20. Heat map analysis showing gene expression profiles of liver resident macrophages/Kupffer cells. The identity of each cluster was assigned by matching the cluster expression profile with established markers. Top differentially expressed genes expressed in each cell type are shown.

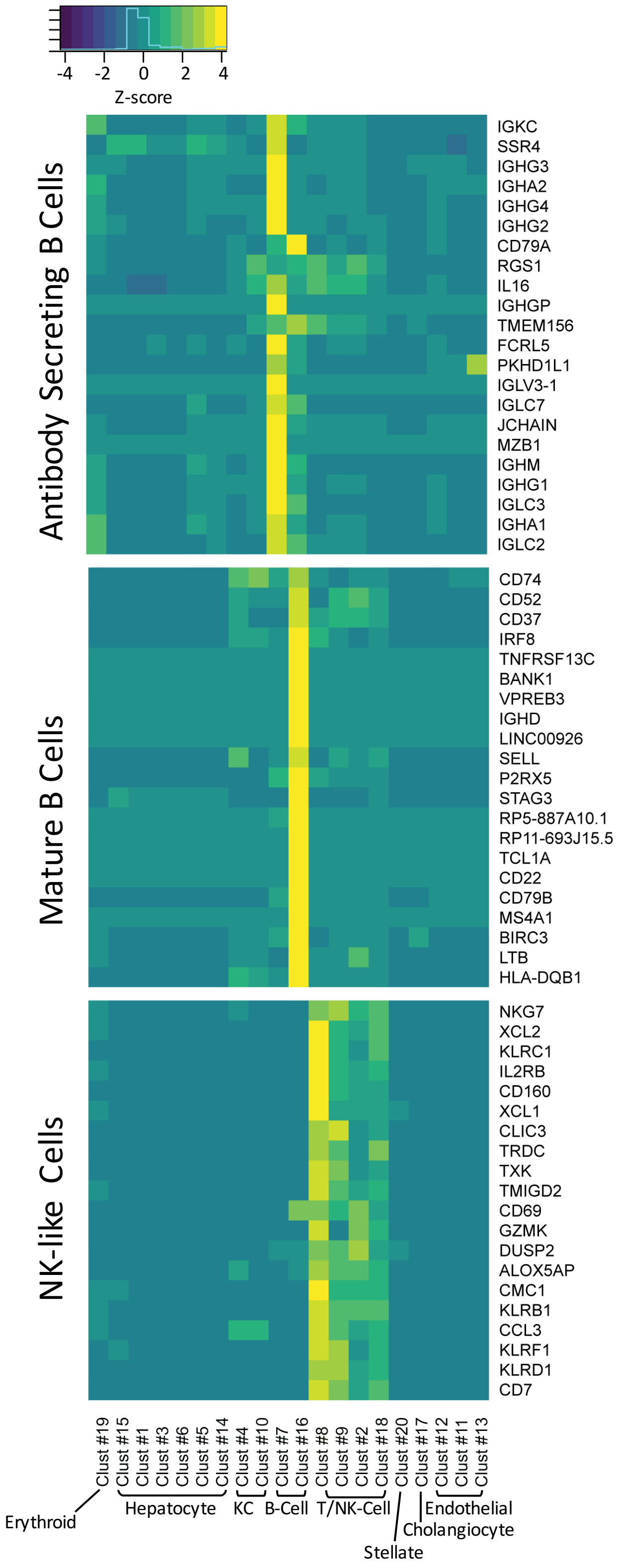

Supplementary Figure 21. Heat map analysis showing gene expression profiles of NK-like and B cells. The identity of each cluster was assigned by matching the cluster expression profile with established markers. Top differentially expressed genes expressed in each cell type are shown.

NK: natural killer

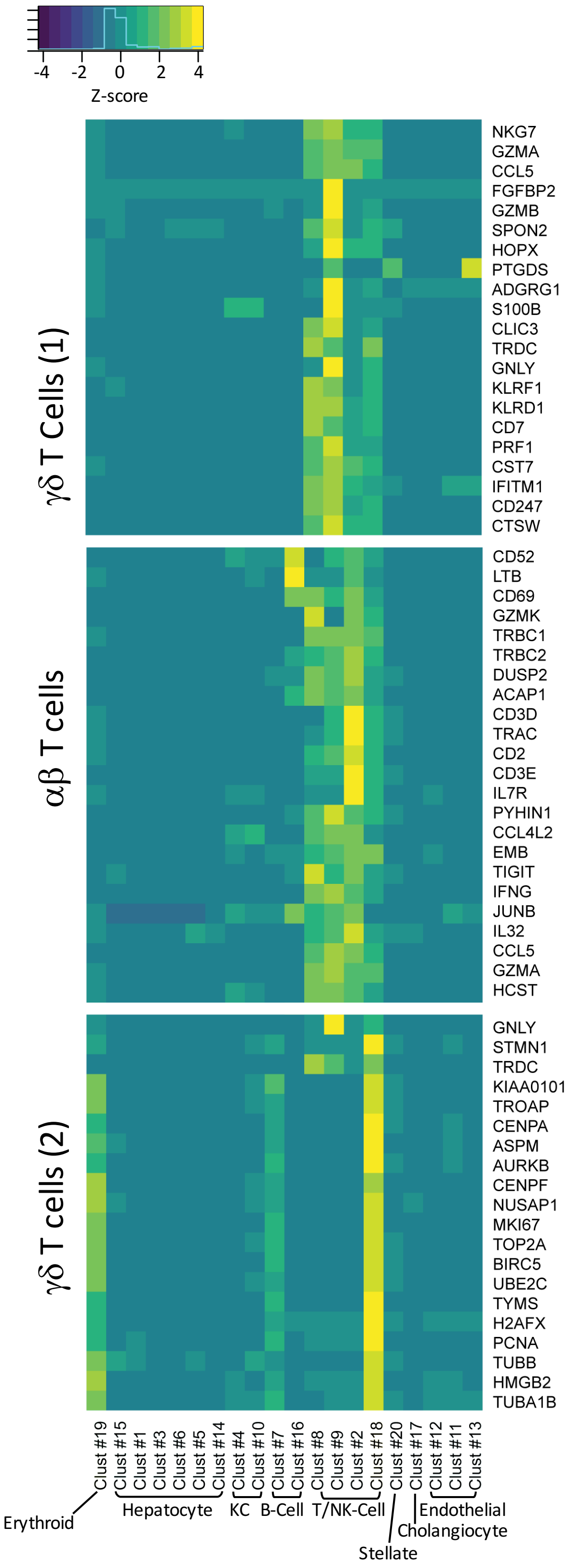

Supplementary Figure 22. Heat map analysis showing gene expression profiles of T cells. The identity of each cluster was assigned by matching the cluster expression profile with established markers. Top differentially expressed genes expressed in each cell type are shown.

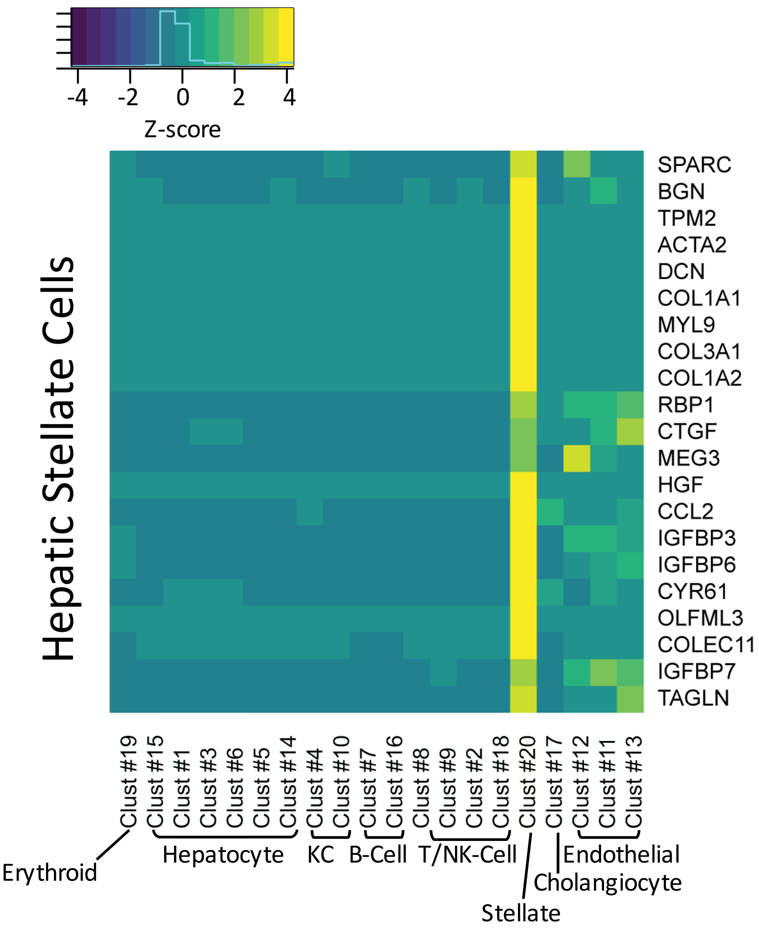

Supplementary Figure 23. Heat map analysis showing gene expression profiles of hepatic stellate cells. The identity of the cluster was assigned by matching the cluster expression profile with established markers. Top differentially expressed genes expressed in each cell type are shown.

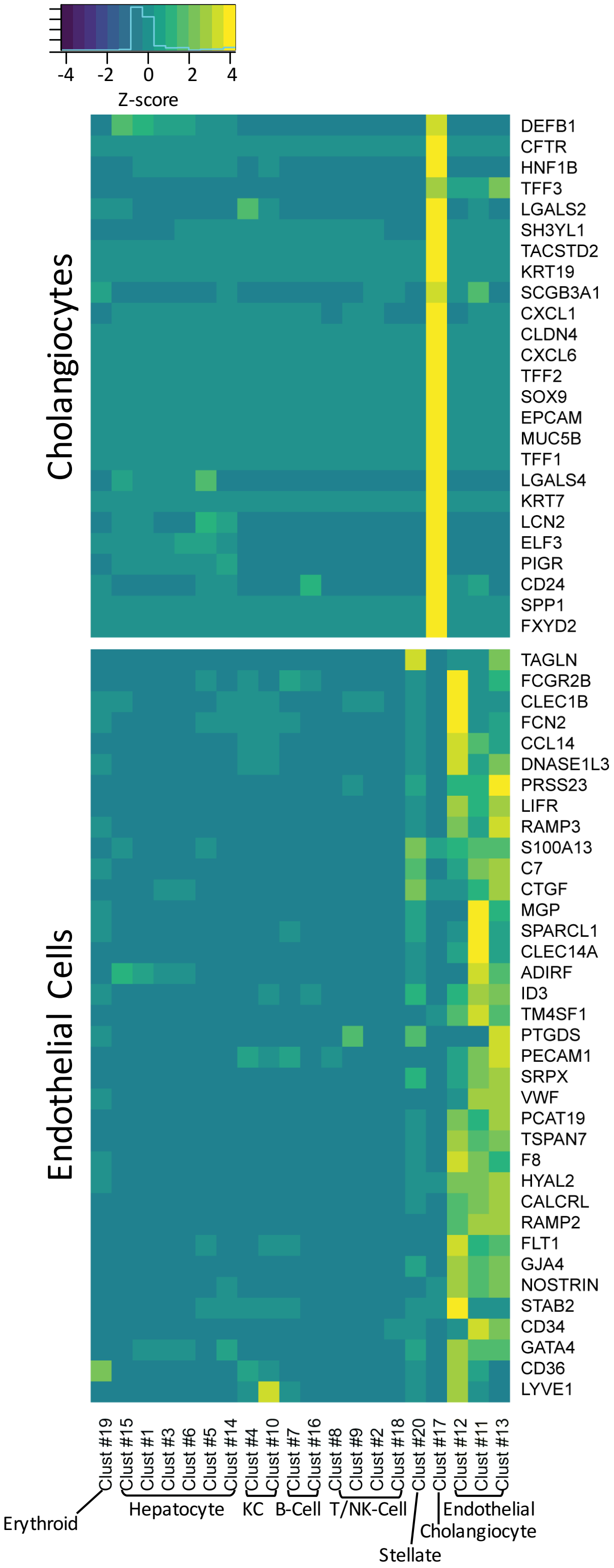

Supplementary Figure 24. Heat map analysis showing gene expression profiles of endothelial cells and cholangiocytes. The identity of each cluster was assigned by matching the cluster expression profile with established markers. Top differentially expressed genes expressed in each cell type are shown.

Estimated Number of Cells
1,771

Mean Reads per Cell	Median Genes per Cell
261,567	179

Sequencing

Number of Reads	463,236,917
Valid Barcodes	98.6%
Reads Mapped Confidently to Transcriptome	70.2%
Reads Mapped Confidently to Exonic Regions	73.0%
Reads Mapped Confidently to Intronic Regions	6.7%
Reads Mapped Confidently to Intergenic Regions	6.4%
Reads Mapped Antisense to Gene	2.3%
Sequencing Saturation	92.4%
Q30 Bases in Barcode	96.8%
Q30 Bases in RNA Read	78.4%
Q30 Bases in Sample Index	95.6%
Q30 Bases in UMI	97.4%

Cells

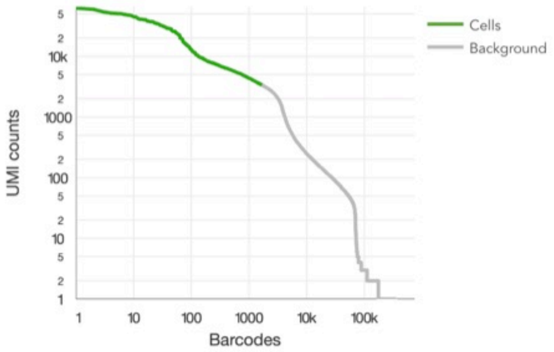

Estimated Number of Cells	1,771
Fraction Reads in Cells	49.0%
Mean Reads per Cell	261,567
Median Genes per Cell	179
Total Genes Detected	18,832
Median UMI Counts per Cell	4,651

Sample

Name	Total Liver Homogenate 1
Description	
Transcriptome	GRCh38
Chemistry	Single Cell 3' v2
Cell Ranger Version	2.0.0

Supplementary Figure 25: 10x Genomics Cell Ranger software summaries of unfiltered data from total liver homogenate 1.

Estimated Number of Cells
5,189

Mean Reads per Cell	Median Genes per Cell
103,549	425

Sequencing

Number of Reads	537,316,704
Valid Barcodes	98.4%
Reads Mapped Confidently to Transcriptome	31.8%
Reads Mapped Confidently to Exonic Regions	33.7%
Reads Mapped Confidently to Intronic Regions	5.9%
Reads Mapped Confidently to Intergenic Regions	5.3%
Reads Mapped Antisense to Gene	2.6%
Sequencing Saturation	85.7%
Q30 Bases in Barcode	96.5%
Q30 Bases in RNA Read	69.0%
Q30 Bases in Sample Index	95.2%
Q30 Bases in UMI	97.1%

Cells

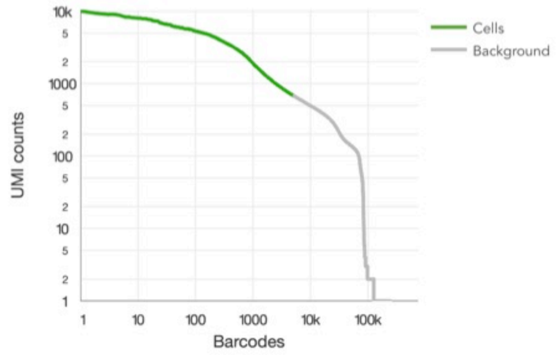

Estimated Number of Cells	5,189
Fraction Reads in Cells	34.0%
Mean Reads per Cell	103,549
Median Genes per Cell	425
Total Genes Detected	19,037
Median UMI Counts per Cell	973

Sample

Name	Total Liver Homogenate 2
Description	
Transcriptome	GRCh38
Chemistry	Single Cell 3' v2
Cell Ranger Version	2.0.0

Supplementary Figure 26: 10x Genomics Cell Ranger software summaries of unfiltered data from total liver homogenate 2.

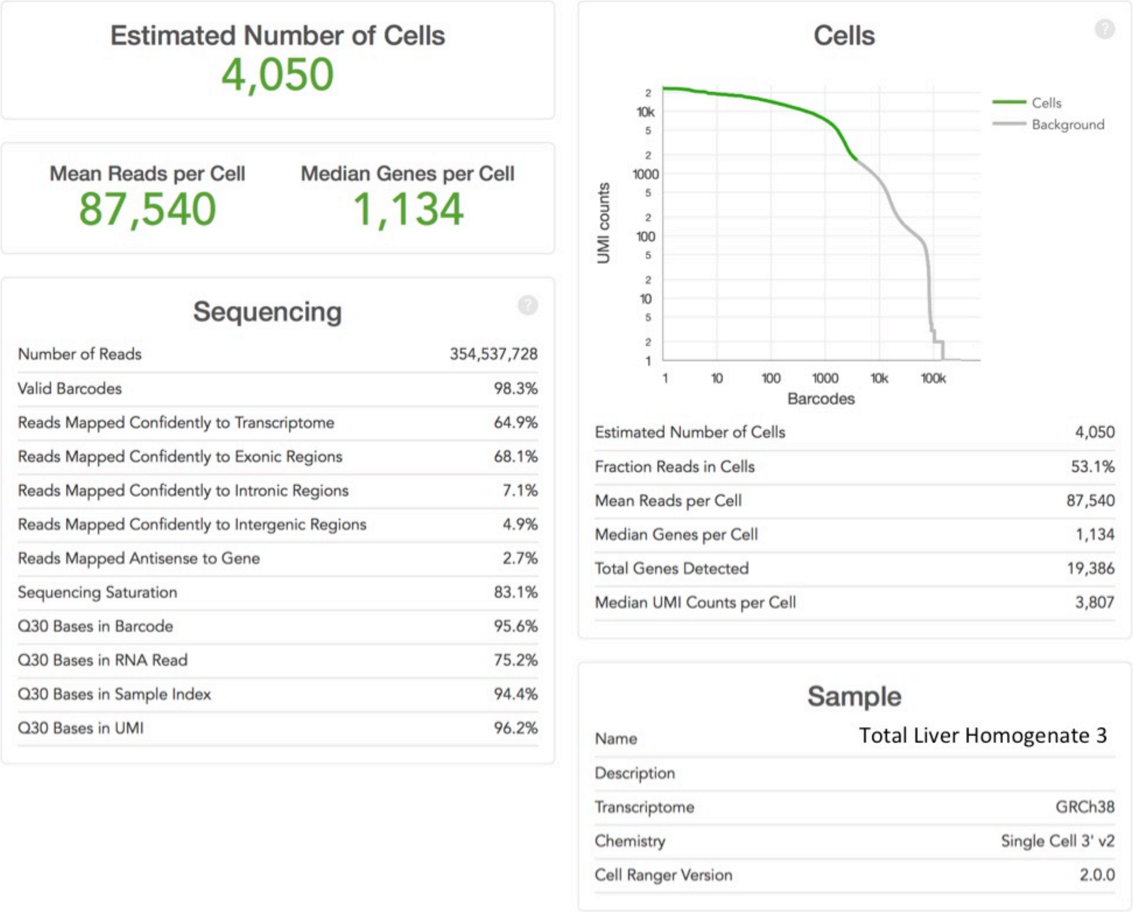

Supplementary Figure 27: 10x Genomics Cell Ranger software summaries of unfiltered data from total liver homogenate 3.

Estimated Number of Cells
1,974

Mean Reads per Cell	Median Genes per Cell
232,564	954

Sequencing

Number of Reads	459,081,940
Valid Barcodes	98.3%
Reads Mapped Confidently to Transcriptome	74.9%
Reads Mapped Confidently to Exonic Regions	77.5%
Reads Mapped Confidently to Intronic Regions	4.4%
Reads Mapped Confidently to Intergenic Regions	9.3%
Reads Mapped Antisense to Gene	2.0%
Sequencing Saturation	84.6%
Q30 Bases in Barcode	95.6%
Q30 Bases in RNA Read	75.8%
Q30 Bases in Sample Index	94.4%
Q30 Bases in UMI	96.2%

Cells

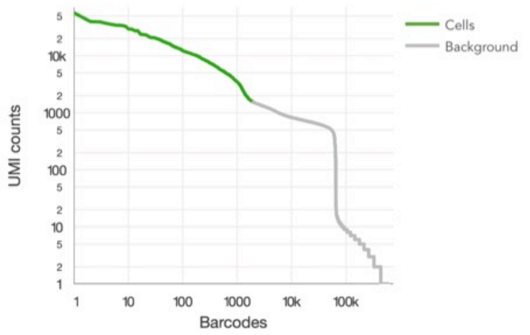

Estimated Number of Cells	1,974
Fraction Reads in Cells	19.2%
Mean Reads per Cell	232,564
Median Genes per Cell	954
Total Genes Detected	19,380
Median UMI Counts per Cell	3,514

Sample

Name	Total Liver Homogenate 4
Description	
Transcriptome	GRCh38
Chemistry	Single Cell 3' v2
Cell Ranger Version	2.0.0

Supplementary Figure 28: 10x Genomics Cell Ranger software summaries of unfiltered data from total liver homogenate 4.

Estimated Number of Cells
5,461

Mean Reads per Cell	Median Genes per Cell
68,150	205

Sequencing

Number of Reads	372,170,107
Valid Barcodes	98.2%
Reads Mapped Confidently to Transcriptome	70.6%
Reads Mapped Confidently to Exonic Regions	73.3%
Reads Mapped Confidently to Intronic Regions	8.3%
Reads Mapped Confidently to Intergenic Regions	8.0%
Reads Mapped Antisense to Gene	2.1%
Sequencing Saturation	84.5%
Q30 Bases in Barcode	96.2%
Q30 Bases in RNA Read	80.6%
Q30 Bases in Sample Index	95.1%
Q30 Bases in UMI	96.7%

Cells

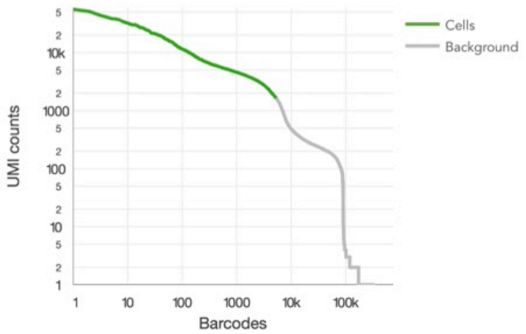

Estimated Number of Cells	5,461
Fraction Reads in Cells	54.2%
Mean Reads per Cell	68,150
Median Genes per Cell	205
Total Genes Detected	20,314
Median UMI Counts per Cell	3,036

Sample

Name	Total Liver Homogenate 5
Description	
Transcriptome	GRCh38
Chemistry	Single Cell 3' v2
Cell Ranger Version	2.0.0

Supplementary Figure 29: 10x Genomics Cell Ranger software summaries of unfiltered data from total liver homogenate 5.

Liver Sample Number	Age	Sex	Race	BMI	Caudate Weight (g)	Cause of Death	Mechanism	Serologies	Smoking	Alcohol-use	Drug-use
1	44	M	N/A	30.5	NA	Head Trauma	Fall	CMV	1/2 pack/d over 40y	daily (alcoholic)	marijuana - infrequent
2	65	M	White	31.7	24.89	Head Trauma	Unknown	CMV, EBV	not reported	not reported	not reported
3	41	F	White	37.1	41.89	Brain aneurysm	aneurysmal rupture	EBV	1/2 pack/d over 25y	socially	not reported
4	21	M	White	28.1	23.49	Anoxia	Drug overdose	N/A	1/2 pack/d over 10y	heavy	medicinal marijuana, heroin
5	26	M	Black	28.4	30.47	Stroke	Dural arteriovenous fistula	EBV	not reported	not reported	not reported

All donors meet NDD criteria

Supplementary Table 1: Neurologically deceased liver donor details. BMI: Body mass index, NDD: Neurologically deceased donor, CMV: Cytomegalovirus, EBV: Epstein-Barr virus.
