# Supplementary material for: Single cell RNA sequencing of human liver reveals distinct intrahepatic macrophage populations
Source: Nat Commun. 2018 Oct 22;9:4383. doi: 10.1038/s41467-018-06318-7 (PMC6197289; doi:10.1038/s41467-018-06318-7)
Supplement: Supplementary file 3 — Description of Additional Supplementary Files [file 41467_2018_6318_MOESM3_ESM.docx]

**Description of Additional Supplementary Files**

**File Name:** Supplementary Data 1

**Description:** Ranked list of all significantly differentially expressed genes in 20 hepatic cell clusters ranked by fold change in expression.

**File Name:** Supplementary Data 2

**Description:** Top 20 significantly differentially expressed genes in each of 20 hepatic cell clusters (ranked by P-value).

**File Name:** Supplementary Data 3

**Description:** Raw data for correlational study of mouse *vs* human liver.

**File Name:** Supplementary Data 4

**Description:** Human hepatocyte clusters GSVA analysis raw data.

**File Name:** Supplementary Data 5

**Description:** Ranked list from pairwise comparison of AFP^+^ vs AFP^-^ hepatocytes.

**File Name:** Supplementary Data 6

**Description:** Ranked list of all significantly differentially expressed genes in T and NK-like sub-clusters (Ranked by fold change).

**File Name:** Supplementary Data 7

**Description:** Cell Cycle Marker Genes.
